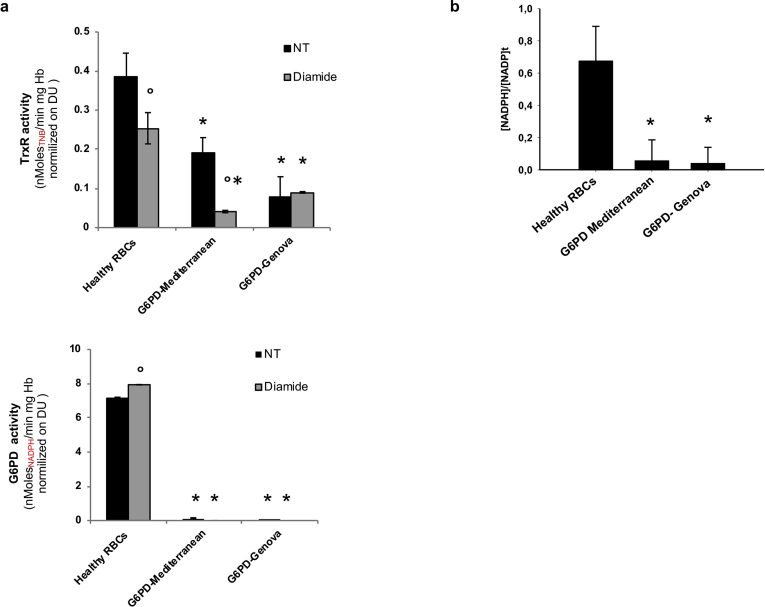# Fyn specifically Regulates the activity of red cell glucose-6-phosphate-dehydrogenase

**DOI:** 10.1016/j.redox.2020.101639

**Published:** 2020-07-11

**Authors:** Matte’ Alessandro, Lupo Francesca, Tibaldi Elena, Di Paolo Maria Luisa, Federti Enrica, Andrea Carpentieri, Piero Pucci, Brunati Anna Maria, Cesaro Luca, Turrini Francesco, Gomez Manzo Saul, Soo Young Choi, Marcial Quino Jaime, Kim Dae Won, Antonella Pantaleo, Xiuli An, Iana Iatcenko, Cappellini Maria Domenica, Forni Gian Luca, De Franceschi Lucia

**Affiliations:** a Dept of Medicine University of Verona and AOUI Verona, Verona, Italy; b Dept of Molecular Medicine, University of Padua, Padua, Italy; c Dept of Chemical Sciences, University Federico II, Naples, Italy; d Dept of Oncology, University of Torino, Torino, Italy; e Laboratorio de Bioquímica Genética, Instituto Nacional de Pediatría, Secretaría de Salud, Mexico City, Mexico; f Institute of Bioscience and Biotechnology, Hallym University, Gangowo-do, South Korea; g Consejo Nacional de Ciencia y Tecnology, Instituto Nacional de Pediatría, Secretaría de Salud, Mexico City, Mexico; h Dept of Physiology, University of Sassari, Sassari, Italy; i School of Life Sciences, Zhengzhou University, Zhengzhou, China; j Laboratory of Membrane Biology, New York Blood Center, New York, NY, USA; k Dept of Medicine, University of Milan, Milan, Italy; l Centro Della Microcitemia e Delle Anemie Congenite, Ospedale Galliera, Genova, Italy

**Keywords:** Red cells, Primaquine, G6PD, Oxidation, Signaling, Tyr, tyrosine, G6PD, glucose 6 phosphate dehydrogenase, RBC, red blood cells, ROS, reactive oxygen species, SFK, Src family kinase, NADP, nicotinamide adenine dinucleotide phosphate, Prx2, peroxiredoxin-2, GSH, glutathione, Phe, phenylalanine, Trp, Tryptophan, HSP, heat shock protein, DTT, dithiothreitol, NEM, N-ethylmaleimide, SDS-PAGE, sodium dodecyl sulphate-polyacrilamide gel electrophoresis

## Abstract

Fyn is a tyrosine kinase belonging to the Src family (Src-Family-Kinase, SFK), ubiquitously expressed. Previously, we report that Fyn is important in stress erythropoiesis. Here, we show that in red cells Fyn specifically stimulates G6PD activity, resulting in a 3-fold increase enzyme catalytic activity (k_cat_) by phosphorylating tyrosine (Tyr)-401. We found Tyr-401 on G6PD as functional target of Fyn in normal human red blood cells (RBC), being undetectable in G6PD deficient RBCs (G6PD-Mediterranean and G6PD-Genova). Indeed, Tyr-401 is located to a region of the G6PD molecule critical for the formation of the enzymatically active dimer. Amino acid replacements in this region are mostly associated with a chronic hemolysis phenotype. Using mutagenesis approach, we demonstrated that the phosphorylation status of Tyr401 modulates the interaction of G6PD with G6P and stabilizes G6PD in a catalytically more efficient conformation. RBCs from *Fyn*^-/−^mice are defective in G6PD activity, resulting in increased susceptibility to primaquine-induced intravascular hemolysis. This negatively affected the recycling of reduced Prx2 in response to oxidative stress, indicating that defective G6PD phosphorylation impairs defense against oxidation. In human RBCs, we confirm the involvement of the thioredoxin/Prx2 system in the increase vulnerability of G6PD deficient RBCs to oxidation. In conclusion, our data suggest that Fyn is an oxidative radical sensor, and that Fyn-mediated Tyr-401 phosphorylation, by increasing G6PD activity, plays an important role in the physiology of RBCs. Failure of G6PD activation by this mechanism may be a major limiting factor in the ability of G6PD deficient RBCs to withstand oxidative stress.

## Highlights

• In red cells, Fyn acts as an oxidative sensor.
• Fyn specifically stimulates G6PD activity by phosphorylating tyrosine (Tyr)-401.
• In Fyn-/-mice, failure of G6PD activation increases red cell vulnerability against oxidation.
• Fyn targeting G6PD protects Prx2 recycling, which is required to remove peroxides.

## Graphical abstract

**Figure:**
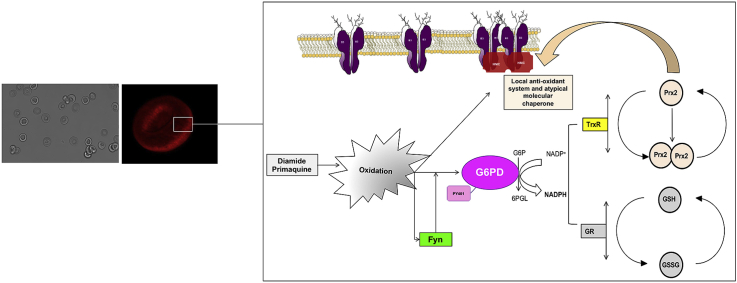

## 1 Introduction

In red cells, G6PD is part of the pentose phosphate pathway (PPP), which is the main source of NADPH. Thus, G6PD is extremely important in red cell homeostasis against cellular stresses such as oxidation associated with the ingestion of fava beans or the exposition to pro-oxidant therapeutic molecules (e.g. primaquine) or severe infections (e.g. sepsis) [[1], [2], [3], [4]]. A worldwide distributed hereditary red cell enzymopathy is the G6PD deficiency [[1], [2], [3], [4]]. The inability of G6PD deficient red cells to be protected against increased reactive oxygen species (ROS) has been mainly related to their incapacity to remove peroxides through the glutathione peroxidase/reductase system [5]. However, the mild hematologic phenotype observed in mice genetically lacking glutathione peroxidase, suggests the possible involvement of other systems such as thioredoxin in controlling ROS levels in red cells [[6], [7], [8]].

In the last two decades, molecular studies have allowed the investigators to characterize and understand G6PD function based on the identification of critical sequence(s) of the protein [4,9]. Indeed, mutations affecting the 380–410 aa stretch correspond to the subunit interface in enzymatically active G6PD. This results in chronic non-spherocytic hemolytic anemia that is rarely transfusion dependent and differs from the more common clinical presentation of G6PD-deficiency as acute hemolysis triggered by ingestion of fava beans or the exposition to pro-oxidant drugs [1,4,9,10]. Active G6PD exists in equilibrium between dimers and tetramers and the association between G6PD subunits is NADP dependent, which might play a role in stabilizing G6PD structure or in catalytic function of the enzyme [2,11]. The importance of the G6PD region in interaction with NADP has been confirmed by mutagenesis studies, which indicate the key role of Tyrosine (Tyr-) 509 and Tyr-401 in interacting with the pyramidal ring of NADP possibly affecting either the protein stability or the protein affinity for structural G6PD [[12], [13], [14]].

Previous studies in liver from rat and in endothelial cell lines have suggested a possible involvement of the Src family kinase (SFK) as up-stream regulator of G6PD [15,16]. Both studies documented the ability of Src kinase to increase G6PD activity [15,16], indicating the importance of post-translational modifications as key events in regulation of G6PD activity.

Among SFK, Fyn has been shown to be activated by ROS, participating to cellular response against oxidation [17,18]. Recently, we report a novel role of Fyn in response to stress erythropoiesis, indicating the importance of Fyn in cell signaling against oxidation [19]. Since Fyn^-/-^ mice display a mild hemolytic anemia with slight but significant increase in the reticulocyte count (Table 2S, see also Beneduce et al.) [19], we hypothesize a possible role of Fyn in red cell homeostasis against physiologic oxidative stress related to red cell survival in the peripheral circulation.

In the present study, we tested whether G6PD activity might be modulated by Fyn kinase in red cells. We identify residue Tyr401 as specific target of Fyn. Using mutagenesis approach, we show that the catalytic efficiency of G6PD towards NADP+ and G6P is strongly increased by Tyr-phosphorylation. We document that red cells from Fyn^-/−^mice are sensitive to primaquine induced intravascular hemolysis similar to that observed in G6PD deficient patients. Finally, we link the delayed recycling of the reduced Prx2, a key anti-oxidant system in red cells, with G6PD deficiency due to either G6PD mutations or Fyn absence. This indicate Prx2 as key NADPH dependent anti-oxidant additional to glutathione peroxidase preventing hemolysis induced by oxidation.

## 2 Methods

### 2.1 Design of the study

The Institutional Animal Experimental Committee of University of Verona (CIRSAL) and the Italian Ministry of Health approved the experimental protocols (prot. 56DC9.21), following the European directive 2010/63/EU and the FELASA guidelines and recommendations. Two-months old female wild-type (WT) and Fyn^-/-^ mice were studied [19]. Female animals were used due to gender difference in hematologic response to erythropoietin [20]. Whenever indicated mouse red cells were *in vitro* treated with diamide as previously reported [21]. Primaquine was administrated at the dosage of 25 mg/Kg by a single intraperitoneal injection and mice were sacrificed at day 7, as previously described [22].

G6PD deficient and healthy controls were matched by age, gender and ethnic background. Each patient was informed on the ongoing study and written informed consent was obtained. Blood was collected in EDTA tube and immediately processed. The study was approved by the Ethical Committee of the Azienda Ospedaliera Integrata of Verona (Italy) and informed consent was obtained from patients and healthy controls (Ethical approval #FGRF13IT).

### 2.2 Hematologic parameters

Details are reported online as Supplemental Methods [19,23].

### 2.3 Immunoblot and immunoprecipitation assays

Details are reported as Supplemental Methods [21,[24], [25], [26]].

### 2.4 Measurements of band 3 clusterization, membrane associated hemichromes and erythroid microparticles

Details are reported online as Supplemental Methods [21,[24], [25], [26]].

### 2.5 G6PD and thioredoxin reductase activities

G6PD and Thiroredoxin reductase activities were carried out in mouse and human red cells. Details are reported as Supplemental Methods [27,28].

### 2.6 NADPH and total NADP determination

Details are reported in Supplemental Methods [29].

### 2.7 Catalase activity

Details are reported in Supplemental Methods [30].

### 2.8 GSH activity

GSH activity was determined as previously reported by Ayi et al. [31].

### 2.9 Protein identification and G6PD phospho-mapping

Peptides mixtures were analyzed by LC-MSMS on a 6520 Accurate-Mass Q-Tof LC/MS System (Agilent Technologies, Palo Alto, CA, USA) equipped with a 1200 HPLC System and a chip cube (Agilent Technologies). Details are reported in Supplemental Methods.

### 2.10 G6PD mutants and kinetic studies

Details are reported as Supplemental Methods [12].

### 2.11 Statistical analysis

Data were analyzed using either *t*-test or one-way ANOVA (Dunnet’s test) for longitudinal studies or one-way ANOVA with Bonferroni correction for multiple comparisons or two-way ANOVA with Bonferroni correction for multiple comparisons. A difference with a p< 0.05 was considered significant.

## 3 Results

### 3.1 Fyn activation in response to oxidation specifically targets Tyrosine-401 residue on G6PD

**Fig. 1:**
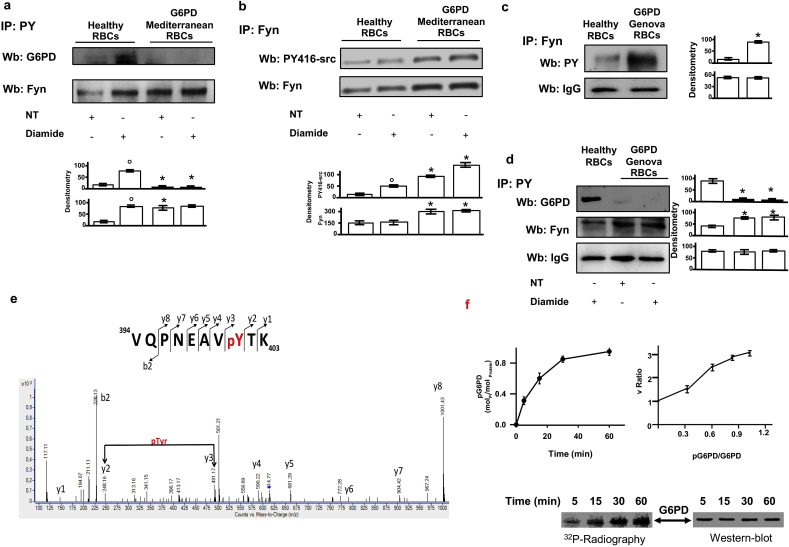
**In human red cells exposed to oxidation, G6PD is Tyrosin-phosphorylated by Fyn, which target Tyr401 residue on G6PD**. **(a)** Cytosol fraction from red cells of healthy and G6PD-Mediterranean subjects treated with or without (NT: non-treated) diamide underwent immunoprecipitation with specific anti-phospho-Tyrosine antibodies (IP: PY) and then used for Western-blot (Wb) analysis with either anti-G6PD or anti-Fyn antibodies. Twin colloidal Commassie stained gels as well as catalase in IP supernatant were used as loading controls run (see 1Sa). One representative gel from other 4 with similar results is presented. **Lower panel.** Relative quantification of immunoreactivity for Fyn or PY catalase (densitometric intensity was relative to catalase). Data are presented as mean ± SD (*n* = 4; *p< 0.05 compared to WT; ° p<0.05 compared to non-treated red cells). **(b)** Cytosol fraction from red cells of healthy and G6PD-Mediterranean subjects treated with or without (NT: non-treated) diamide underwent Western-blot (Wb) analysis with either anti-G6PD or anti-Fyn antibodies. Catalase in IP supernatant was used as protein loading control (see 1Sb). One representative gel from other 4 with similar results is presented. **Lower panel.** Relative quantification of immunoreactivity for Fyn or PY (densitometric intensity was relative to catalase). Data are presented as mean ± SD (*n* = 4; *p< 0.05 compared to healthy red cells; ° p<0.05 compared to non-treated red cells). **(c)** Cytosol fraction from red cells of healthy and G6PD-Genova subjects exposed to diamide underwent immunoprecipitation respectively with specific anti-phospho-Tyrosine antibodies (IP: PY) or anti-Fyn and then used for Western-blot (Wb) analysis with anti-phospho-Tyr-416 Src antibody to detect active form of Fyn (PY420) and anti-Fyn. One representative gel from other 4 with similar results is presented. Catalase in IP supernatant was used as loading control (Fig. 1Sc). **Right panel.** Relative quantification of immunoreactivity for active and total Fyn (densitometric intensity was relative to catalase). Data are presented as mean ± SD (*n* = 4; *p< 0.05 compared to healthy red cells; ° p<0.05 compared to non-treated red cells). **(d)** Cytosol fraction from red cells of healthy and G6PD Genova subjects treated with or without (NT: non-treated) diamide underwent immunoprecipitation with specific anti-phospho-Tyrosine antibodies (IP: PY) and then used for Western-blot (Wb) analysis with either anti-G6PD or anti-Fyn antibodies. Catalase in IP supernatant was used as loading control (Fig. 1Sd). One representative gel from other 4 with similar results is presented. **Right panel.** Relative quantification of immunoreactivity for Fyn or PY (densitometric intensity was relative to catalase). Data are presented as mean ± SD (*n* = 4; *p< 0.05 compared to healthy red cells; ° p<0.05 compared to non-treated red cells). **a-d** Data are presented as mean ± SD; *p< 0.05 compared to healthy red cells; ° p<0.05 compared to non-treated red cells by two-way ANOVA with Bonferroni correction for multiple comparisons. **(e)** Fragmentation spectrum of the G6PD peptide 395–403 from the LC-MS/MS analysis of an immuno-enriched sample of phospho-G6PD from diamide treated healthy red cells red cells following tryptic hydrolysis. The figure shows the occurrence of pTyr at position 401. **(f) Upper left panel**. Time course of wild type G6PD phosphorylation by Fyn. Values are the mean ± SEM of four determinations performed at each time point. **Upper right panel**. G6PD activity (reported as % of fold change, y-axis) of equivalent amounts of G6PD previously phosphorylated by Fyn at different time points (reported as G6PD-P/G6PD_TOT_ ratio). Each value is the mean ± SEM of four determinations. **Lower panel.** One representative gel from 4 with similar results is presented and it refers to the time course of phosphorylation of wild type G6PD by Fyn (upper left panel). (For interpretation of the references to colour in this figure legend, the reader is referred to the Web version of this article.)

Here, we studied red cells from G6PD-Mediterranean and G6PD Genova, the latter is a part of G6PD-deficincy with chronic hemolysis [1]. We used diamide as exogenous oxidant to promote severe red cells oxidative damage [28]. Since we previously shown that Fyn is important in response to oxidative stress in maturating erythroid cells, we evaluated the impact of diamide on Fyn activation. As shown in Fig. 1a, diamide significantly increased the amount of phospho-Tyrosin (Tyr)-Fyn in healthy red cells, whereas in G6PD we observed higher amount of phospho-Tyr-Fyn in both vehicle and diamide treated erythrocytes. This latter was slightly higher than that observed in vehicle treated G6PD-Mediterranea erythrocytes. Noteworthy, we found that diamide promoted Tyr-phosphorylation of G6PD in healthy red cells, but not in G6PD-Mediterranea erythrocytes (Fig. 1a and 1b, Fig. 1Sa, 1Sb). The phosphorylation state of Fyn was probed with anti-phospho-Tyr-416 Src antibody to detect active form of Fyn (PY420) in red cells from both healthy and G6PD Mediterranean patients. As shown in Fig. 1b, we found activation of Fyn in response to diamide in red cells from both control and G6PD subjects, indicating Fyn as oxidative sensor in red cells. We then evaluated Fyn active form in red cells from G6PD-Genova. As shown in Fig. 1c, Fyn was more active in G6PD-Genova than in heathy controls (Fig. 1Sc). In addition, we found again the lacking of Tyr-G6PD form in G6PD-Genova in response to diamide compared to diamide induced increased Tyr-phosphorylation observed in healthy erythrocytes (Fig. 1d and Figure 1Sd). To evaluate the presence of possible Fyn target(s) on G6PD, we carried out an extensive bioinformatic analysis of putative Src/Fyn target sites on G6PD. Analysis of the of G6PD sequence for Src-directed peptides with a high threshold score >2.4 identified 7 Tyr-residues on mouse G6PD and 5 Tyr-residues on human G6PD. The analysis restricted to Fyn-specific target sequences indicated two tyrosine residues: Tyr-147 (score 2.87) and Tyr-401 (score 5.163) as possible modification sites (Fig. 1Se). We then generated immuno-enriched samples of phospho-G6PD from diamide treated red cells, which underwent to LC-MS/MS analysis. We identified Tyr-401 to be Tyr-phosphorylated *in vivo* in phospho-G6PD enriched samples (Fig. 1e). Noteworthy, Tyr-401 is located in the COOH terminus in a protein area involved in the interaction with the pyramidal ring of NADP+ [12,13]. To validate our finding, we generated recombinant G6PD, which was incubated *in vitro* with either recombinant Fyn or Lyn or Syk. As shown in Fig. 1f, G6PD activity (right panel) was increased by Fyn phosphorylation (left panel). Whereas, no change in G6PD phosphorylation state was observed in presence of either Lyn or Syk kinase (data not shown). Recombinant G6PD was incubated with Fyn, digested with trypsin and the resulting peptide mixture was analyzed by MLC-MS/MS. Manual inspection of the fragmentation spectra of the 394–403 peptide confirmed Tyr-401 as specific target of Fyn (data not shown). Collectively these data support the novel functional link between G6PD and Fyn, specifically targeting Tyr401 residue in response to oxidative stress.

### 3.2 Tyr401 on G6PD plays a key role in modulation of G6PD activity

**Fig. 2:**
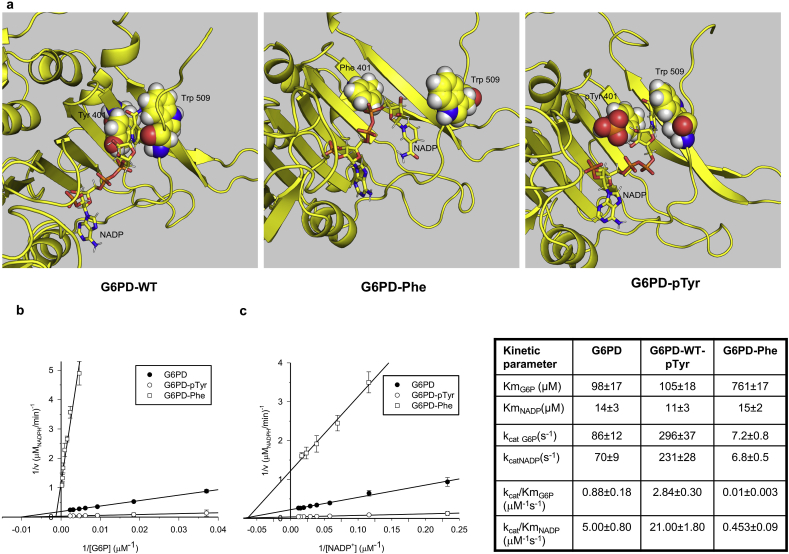
**Tyr 401 on G6PD is important for G6PD conformation and activity**. **(a)** Molecular dynamics simulations of G6PD dimer (wt, Y401F, pTyr401) NADP is represented as sticks, Tyr 401 and Trp 509 as spheres. For clarity, only one monomer is represented**. (b**–**c)** Determination of kinetic parameters of wt, phosphorylated and Phe mutant G6PD. Linewaver-Burk plots of enzyme activity of G6PD WT (●), phosphorylated-G6DP (G6PD-pTyr) (○) and G6DP-Phe (□) for the determination of the steady-state kinetic parameters versus the two substrates: G6P in (b) (fixed and saturating [NADP^+^] = 86uM) and NADP+ (in **c**) (saturating [G6P] = 0.54 mM with wt-enzyme and 4.3 mM with Phe-mutant enzyme); human recombinant [G6PD] = 2 nM. **Right panel.** Kinetic parameters calculated from the Lineweaver-Burk plots (**b,c**).

To understand the impact of Tyr401, located in the structural NADP + -binding site, on G6PD function, we performed a 20ns molecular dynamic simulation of the G6PD dimer with structural NADP, Tyr401 being replaced by Phe or pTyr when required (Fig. 2a). Structural NADP occupied a crevice located between the C-terminus and β-sheet, the nicotinamide ring being stabilized by Trp509 and Tyr401. The binding of NADP + to this structural binding site, close to the dimer interface, is known to affect the stability of the molecule that is essential also for an efficient catalytic site [[12], [13], [14]]. By replacing Tyr401 with Phe, the C-terminal region moved away from the β-sheet, thereby inducing displacement of Trp509 and loss of interaction with the nicotinamide ring, ultimately resulting in the exit of the NADP^+^ molecule out of the crevice. On the other hand, the presence of the phosphate on the Tyr401 stabilized the C-terminal region and the nicotinamide ring remained locked up between Trp509 and pTyr401. Interestingly, in the simulation of the G6PD-wt dimer only one monomer maintained the C-terminal sequence close to the β-sheet and the nicotinamide ring of NADP blocked between Trp509 and Tyr401, while for G6PD-pTyr401 this occurred in both monomers. This seemed to suggest that the phosphorylation of Tyr401 could stabilize the dimer by maintaining structural NADP in place.

To investigate the effect of Tyr401 phosphorylation on G6DP enzyme activity, we determined K_m_, an index of the substrate's affinity for the enzyme, k_cat_, the catalytic rate constant (or turnover number), and the catalytic efficiency (k_cat_/K_m)_, which determines the enzyme activity under not saturating concentration of substrate, of human recombinant G6PD and its Fyn-phosphorylated form for the substrates G6P and NADP^+^. We then generated a G6PD-Phe mutant to evaluate the role of Tyr401 on enzyme activity (Fig. 2Sa, b). We compared the kinetic behavior of the mutant G6PD-Phe with that of the wild type G6PD-Tyr. The Lineweaver-Burk plots for the three types of recombinant G6PD and the calculated kinetic parameters (Fig. 2b 2c and related kinetic parameters on right panel, respectively) clearly demonstrated how the phosphorylation of Tyr401 increased the k_cat_ values for both the substrates and, consequently, the catalytic efficiency of G6PD itself (k_cat_/K_m_) by three-fold, exhibiting no effect on Km values. Moreover, the replacement of Tyr401 with Phe401 strongly reduced the k_cat_ values for both NADP^+^ and G6P by about 90% as compared with wt-G6PD. This substitution also decreased the affinity for the G6P, with K_m_ values increasing by eight-fold, while the affinity for NADP^+^ did not vary significantly. As a result, the catalytic efficiency (k_cat_/K_m_) of G6PD-Phe was also strongly reduced relative to that of the wild-type enzyme. These data indicate that the phosphorylation status of Tyr401 plays an important role in modulating the interaction of G6PD with G6P and in stabilizing G6PD in a catalytically more efficient conformation. Our data suggest that the absence of Fyn might simulate a G6PD deficiency-like hematologic phenotype.

### 3.3 Red cells genetically lacking Fyn show increased sensitivity to physiologic oxidation with generation of hemichromes and band 3 clusterization

**Fig. 3:**
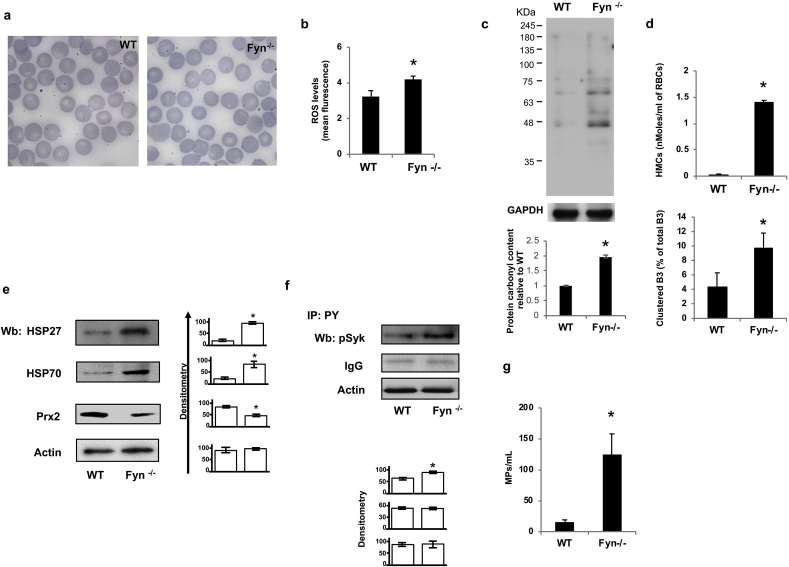
**Fyn**^**-/-**^**mouse red cells display increased ROS levels, hemichromes bound to the membrane and band 3 clusterization promoting the release of erythroid microparticles**. **(a)** Morphology of red cells in May-Grunwald-Giemsa blood smears from wild-type (WT) and Fyn^-/-^ mice. Red cells were imaged under oil at 100X magnification using a Panfluor objective with 1.30 numeric aperture on a Nikon Eclipse DS-5M camera and processed with Digital Slide (DS-L1) Nikon. One representative image from 8 taken for each mouse strains with similar results is presented. **(b)** Reactive oxygen species (ROS) levels in red cells from wild-type (WT) and Fyn^-/-^ mice. Data are presented as means ± SD (n = 6 from each strain); *p<0.05 compared to WT. **(c) Upper panel.** wild-type (WT) and Fyn^-/−^mouse red cell membrane carbonylated proteins (1ug) were detected by treating with DNPH and blotted with anti-DNP antibody. GAPDH was used as protein loading control. **Lower panel.** Quantification of band area was performed by densitometry and expressed as % of wild-type. Data are expressed as means ± SD (*n* = 3 from each strains); *p<0.05 compared to WT.**(d) Upper panel.** Hemichromes (HMCs) bound to the membrane of red blood cells (RBCs) from wild-type (WT) and Fyn^-/-^ mice. Data are presented as means ± SD (*n* = 6); *p<0.05 compared to WT. **Lower panel.** Percentage of band 3 clusters in erythrocytes from wild-type (WT) and Fyn^-/-^ mice. Data are presented as means ± SD (*n* = 6); *p<0.05 compared to WT. **(e)** Western-blot (Wb) analysis with specific antibodies against heat shock proteins (HSP) 27 and 70, peroxiredoxin-2 (Prx2) of red cells membrane from wild-type (WT) and Fyn^-/-^ mice. One representative gel out of 6 with similar results is presented. Actin was used as protein loading control. Densitometric analysis of immunoblots is shown in bar graph (right panel). Data are presented as means ± SD (*n* = 6 from each strains); *p<0.05 compared to WT. **(f)** Red cell ghosts from wild-type (WT) and Fyn^-/-^ underwent immunoprecipitation with specific anti-phospho-Tyrosine antibodies (IP: PY) and then used for Western-blot (Wb) analysis with specific anti-phospho-Syk (pSyk) antibody. IgG and actin were used as loading controls. One representative gel out of 4 with similar results is presented. **Lower panel.** Relative quantification of immunoreactivity for pSyk, IgG and actin. Data are presented as mean ± SD (*n* = 4; *p< 0.05 compared to WT). **(g)** Quantification of microparticles (MPs) from red cells of wild-type (WT) from wild-type (WT) and Fyn^-/-^ mice. Data are presented as means ± SD (*n* = 6); *p<0.05 compared to WT. **a-f.** Data are presented as means ± SD (*n* = 6); *p<0.05 compared to WT by Student’s t-test.

Fyn^-/-^ mice display a mild hemolytic anemia with slight but significant increase in reticulocytes (Table 2S, see also Beneduce et al.) [19]. Although no major abnormalities in red cell morphology were found in Fyn^-/-^ mice (Fig. 3a), we observed higher ROS levels in Fyn^-/-^ mouse red cells when compared to wild-type erythrocytes (Fig. 3b) and increased red cell membrane protein oxidation as determined by the quantification of protein carbonyl groups (Fig. 3c). This was associated with increased hemichromes bound to the membrane and band 3 clusterization in absence of Fyn compared to wild-type red cells (Fig. 3d). The expression of anti-oxidant systems such as peroxiredoxin-2 (Prx2), super-oxide dismutase (SOD-1) as well as of classic chaperones such as heat shock protein 27 and 70 (HSP) was similar in cytosol fraction from red cells of both mouse strains (Fig. 3Sa). Whereas, we observed increased membrane translocation of HSP27 and HSP70, indicating he presence of membrane oxidation in Fyn^-/-^ mouse erythrocytes (Fig. 3e). Noteworthy, the amount of Prx2 bound to the membrane was reduced in Fyn^-/-^ mouse red cells, in agreement with our previous reports showing competition between Prx2 and hemichromes for band 3, their docking site [21,32,33].

Since we previously reported that oxidative stress activates Syk kinase, which promotes band 3 phosphorylation, we evaluated Syk activity in Fyn^-/-^ mouse erythrocytes [24,26,28,34,35]. As shown in Fig. 3f, Syk was more active in Fyn^-/-^ mouse red cells than in wild-type erythrocytes. This was associated with increased release of erythroid microparticles in agreement with our previous reports in red cells from patients with either β-thalassemia or G6PD deficiency exposed to exogenous oxidation (Fig. 3g) [21,26,28,[34], [35], [36]].

### 3.4 Fyn^-/-^ mouse red cells display increased susceptibility to diamide induced oxidation with associated reduced G6PD activity

**Fig. 4:**
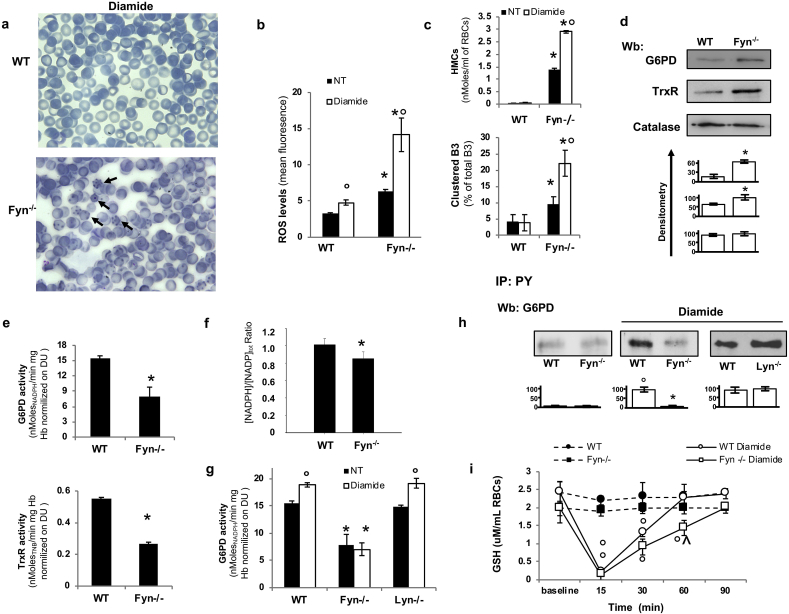
**In Fyn**^**-/-**^**mouse red cells, diamide induces hemoglobin oxidation and severe red cell membrane damage, related to impaired G6PD activity**. **(a)** Morphology of diamide (mM) treated red cells in May-Grunwald-Giemsa blood smears from wild-type (WT) and Fyn^-/-^ mice. The black arrows indicate clusters of oxidized hemoglobin in Fyn^-/-^ mouse red cells. Red cells were imaged under oil at 100X magnification using a Panfluor objective with 1.30 numeric aperture on a Nikon Eclipse DS-5M camera and processed with Digital Slide (DS-L1) Nikon. One representative image out of 4 taken for each mouse strains with similar results is presented. **(b)** Reactive oxygen species (ROS) levels in wild-type (WT) and Fyn^-/-^ mouse red blood cells (RBCs) with (2 mM) or without diamide (non-treated: NT). Data are presented as means ± SD (n = 6 from each strain); *p<0.05 compared to WT; °p<0.05 compared to NT. **(c) Upper panel.** Hemichromes (HMCs) bound to the membrane of wild-type (WT) and Fyn^-/-^ mouse red blood cells (RBCs) with (2 mM) or without diamide (non-treated: NT). Data are presented as means ± SD (*n* = 6); *p<0.05 compared to WT. **Lower panel.** Percentage of band 3 clusters in wild-type (WT) and Fyn^-/-^ mouse red blood cells (RBCs) with (2 mM) or without diamide (non-treated: NT). Data are presented as means ± SD (*n* = 6); *p<0.05 compared to WT. **(d) upper panel.** Western-blot (Wb) analysis with specific antibodies against glucose 6 phosphate dehydrogenase (G6PD) or thioredoxin reductase (TrxR) of red cells from wild-type (WT) and Fyn^-/-^ mice. Catalase was used as loading control. One representative gel out of 6 with similar results is presented. Lower panel. Densitometric analysis of immunoblots is shown in bar graph. Data are presented as means ± SD (*n* = 6 from each strains); *p<0.05 compared to WT. **(e)** Activity of glucose 6 phosphate dehydrogenase (G6PD) and thioredoxin reductase (TrxR) in red cell lysates from wild-type (WT) and Fyn^-/−^mice. Data are presented as means ± SD (*n* = 6 from each strains); *p<0.05 compared to WT. **(f)** NADPH/NADP_total_ ratio in wild-type (WT) and Fyn^-/-^ mice. Data are presented as means ± SD (*n* = 6 from each strains); *p<0.05 compared to WT. [NADP_total_]/[Hb] ratios were not significantly different when WT was compared to Fyn^-/-^ ([NADP_total_]/[Hb]: (1.51 ± 0.31) x 10^-3^ and (1.80 ± 0.46) x 10^-3^, for WT and Fyn^-/-^ mice, respectively). **(g)** Activity of G6PD in red cell lysates from wild-type (WT) and Fyn^-/-^ mice with (2 mM) or without diamide treatment (non-treated: NT). Data are presented as means ± SD (n = 6 from each strain); *p<0.05 compared to WT; °p<0.05 compared to NT. **(h)** Wild-type (WT), Fyn^-/-^ and Lyn^-/-^ mouse cytosol fraction from red cells with or without diamide (non-treated: NT) underwent immunoprecipitation with specific anti-phospho-Tyrosine antibodies (IP: PY) and then used for either Western-blot (Wb) analysis with specific glucose 6 phosphate dehydrogenase (G6PD) antibody or colloidal Commassie staining for protein loading control. Catalase in IP supernatant was used as addition protein loading control (see 4Sb). One representative gel out of 4 with similar results is presented. **Lower panel.** Relative quantification of immunoreactivity for G6PD. Data are presented as mean ± SD (*n* = 4; *p< 0.05 compared to WT; ° p<0.05 compared to non-treated red cells). **b-c-f** *p<0.05 compared to WT; °p<0.05 compared to NT by two-way ANOVA with Bonferroni correction**. d, e, f.** *p<0.05 compared to WT by student’s t-test. **(i)** GSH levels in wild-type (WT), Fyn^-/-^ and Lyn^-/-^ mouse red cells with and without diamide treatment. Data are presented as mean ± SD (*n* = 4); °p<0.02 compared to baseline by one-way ANOVA with Dunnet’s test for longitudinal comparison; ^∧^p<0.05 compared to diamide treated wild-type red cells by two-way ANOVA test with Bonferroni correction for multiple comparisons. (For interpretation of the references to colour in this figure legend, the reader is referred to the Web version of this article.)

To address the question whether Fyn might play a role as oxidative sensor in red cells, we exposed *in vitro* Fyn^-/-^ mouse red cells to diamide, a potent oxidative agent [21,26,28]. Fyn^-/-^ mouse red cells exposed to diamide showed abnormal red cell morphology with Heinz body as well as clustered oxidized hemoglobin bound to the membrane responsible for generating misshaped erythrocytes (Fig. 4a). In Fyn^-/-^ mouse red cells, this was associated with increased (i) ROS; (ii) red cell membrane protein oxidation; (iii) hemichromes bound to the red cell membrane; (iv) band 3 clusterization; (v) Syk activation; and (vi) released of erythroid microparticles compared to wild-type diamide treated red cells (Fig. 3, Fig. 4Sb, c, d). Take together these data driven us to consider a possible perturbation of G6PD function in Fyn^-/-^ mouse red cells, being normal the hemoglobin pattern and red cell membrane protein composition (data not shown).

In Fyn^-/-^ mouse red cells, the expression of G6PD and thioredoxin (TrxR) reductase was significantly increased (Fig. 4d), possibly related to the slight increase in the reticulocytes. However, the activity of both enzymes was markedly reduced in Fyn^-/-^ erythrocytes when compared to wild-type red cells (Fig. 4e). This was associated a significant reduction in NADPH/NADP_tot_ ratio (Fig. 4f). No difference in catalase activity was observed between wild-type and Fyn^-/-^ mouse red cells (Fig. 4Sa).

To better understand the possible role of Fyn in modulation of G6PD functions, we evaluated G6PD activity in red cells from wild-type, Fyn^-/-^ and Lyn^-/-^ mice with and without diamide treatment. Since Lyn is a Src family kinase sharing high level of homology with Fyn [24], we used red cells from Lyn^-/-^ mice to test the specificity of the functional link between Fyn and G6PD activity. As shown in Fig. 4g, G6PD was activated to similar extent in both wild-type and Lyn^-/-^ mouse red cells exposed to diamide, whereas no change in G6PD activity was observed in Fyn^-/-^ mouse red cells. In addition, we found increased amount of Tyr-phosphorylation of G6PD in both wild-type and Lyn^-/-^ mouse red cells exposed to diamide, whereas no change in G6PD Tyr-phosphorylation state was detected in Fyn^-/-^ mouse red cells (Fig. 4h and Figure 4Sb). We then evaluated the regeneration of GSH in wild-type and Fyn^-/-^ mouse red cells exposed to diamide [31]. As shown in Fig. 4i, we observed a delayed in regeneration of GSH in Fyn^-/-^ mouse red cells when compared to wild-type erythrocytes.

**Fig. 5:**
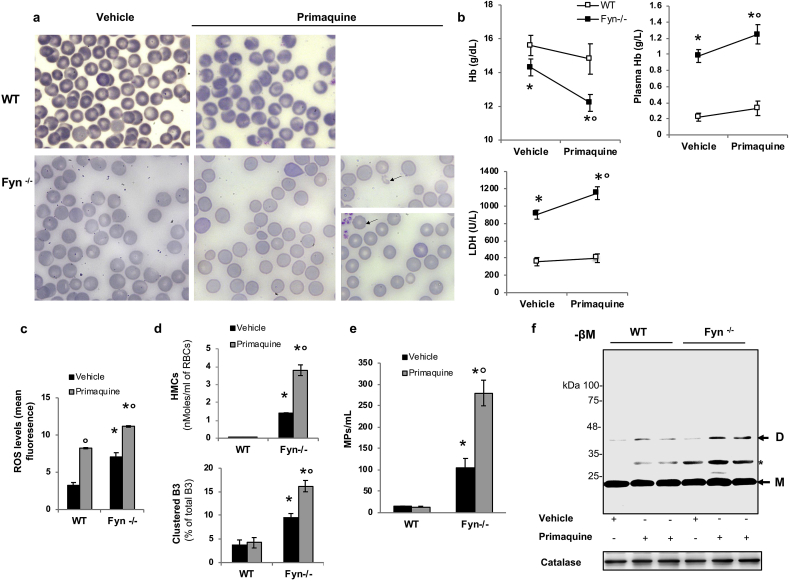
**Primaquine treatment induces acute intravascular hemolytic crisis with severe red cell damage and accumulation of reduced Prx2, a NADPH dependent anti-oxidant system**. **(a)** Morphology of red cells in May-Grunwald-Giemsa blood smears from wild-type (WT) and Fyn^-/-^ mice *in vivo* treated with either vehicle or primaquine. The black arrows indicate red cell ghost and bitted erythrocytes. Red cells were imaged under oil at 100X magnification using a Panfluor objective with 1.30 numeric aperture on a Nikon Eclipse DS-5M camera and processed with Digital Slide (DS-L1) Nikon. One representative image out of 5 for each mouse strains with similar results is presented. **(b)** Hemoglobin, plasma hemoglobin and serum lactate dehydrogenase (LDH) in wild-type (WT) and Fyn^-/-^ mice *in vivo* treated with either vehicle or primaquine. Data are shown as means ± SD (n = 5); *p< 0.05 compared to WT; °p<0.05 compared to vehicle treated animals. **(c)** Reactive oxygen species (ROS) levels in red cells from wild-type (WT) and Fyn^-/-^ mice *in vivo* treated with either vehicle or primaquine. Data are presented as means ± SD (n = 5 from each strain); *p< 0.05 compared to WT; ° p<0.05 compared to vehicle treated animals. **(d) Upper panel.** Hemichromes (HMCs) bound to the membrane of red blood cells (RBCs) from wild-type (WT) and Fyn^-/-^ mice *in vivo* treated with either vehicle or primaquine. Data are presented as means ± SD (n = 5 from each strain); *p< 0.05 compared to WT; ° p<0.05 compared to vehicle treated animals. **Lower panel.** Percentage of band 3 clusters in erythrocytes from wild-type (WT) and Fyn^-/-^ mice *in vivo* treated with either vehicle or primaquine. Data are presented as means ± SD (n = 5 from each strain); *p< 0.05 compared to WT; ° p<0.05 compared to vehicle treated animals. **(e)** Quantification of microparticles (MPs) from wild-type (WT) and Fyn^-/-^ mice *in vivo* treated with either vehicle or primaquine. Data are presented as means ± SD (n = 5 from each strain); *p< 0.05 compared to WT; ° p<0.05 compared to vehicle treated animals. **(f)** Western-blot (Wb) analysis under non-reducing condition (-βM: β-mercaptoethanol) with specific antibodies against peroxiredoxin-2 (Prx2) of red cell cytosol fractions from wild-type (WT) and Fyn^-/-^ mice *in vivo* treated with either vehicle or primaquine. Prx2 monomers (M) and dimers (D) were detected. Catalase was used as loading control. * indicate non-specific signal due to Prx2 binding to hemoglobin chain. One representative gel out of 3 with similar results is presented. Densitometric analysis of immunoblots is shown in bar graph in Supplemental Fig. 5Sa. **b-e.** *p< 0.05 compared to WT; ° p<0.05 compared to vehicle treated animals by two-way ANOVA with Bonferroni correction for multiple comparisons. (For interpretation of the references to colour in this figure legend, the reader is referred to the Web version of this article.)

To further characterize the functional link between Fyn and G6PD activity, we treated wild-type red cells with diamide in the presence or in the absence of PP1-PP2, Src family kinase inhibitors. In wild-type erythrocytes, the diamide induced increased in Tyr-phosphorylation of G6PD was almost completely prevented by PP1-PP2 treatment, while no major difference was observed in Fyn^-/-^ mouse red cells (Fig. 5Sa). This was associated with a significant reduction in diamide induced activation of G6PD in wild-type mouse red cells (Fig. 5Sb). We then evaluated the effect of dithiothreitol (DTT), a thiols-donor on the diamide induced Tyr-phosphorylation of G6PD and the related activation. As shown in Fig. 5Sc, DTT significantly reduced diamide mediated Fyn activation evaluated as both change in Tyr-phosphorylation state of Fyn and in Fyn kinase activity (see also Fig. 5Sd for Fyn kinase activity). In addition, DTT prevented the diamide induced increased Tyr-phosphorylation of G6PD, which was paralleled by marked reduction in diamide induced G6PD activation (Fig. 5Sc, e).

Collectively, these findings further support a role of Fyn in activation of G6PD in response to oxidative stress in mouse red cells.

### 3.5 Fyn^-/-^ mice developed primaquine dependent intravascular hemolysis associated with accumulation of reduced Prx2

Since the anti-malaria drug primaquine has been early linked with acute hemolysis in G6PD deficient subjects, we treated Fyn^-/-^ mice with primaquine [22].

In primaquine treated Fyn^-/-^ mice, we observed: (i) microspherocytosis, Heinz body and the presence of circulating red cell ghosts (Fig. 5a); (ii) acute anemia with intravascular hemolysis as supported by increased plasma hemoglobin and LDH (Fig. 5b); (iii) increased ROS level in circulating red cells associated with high amounts of hemichromes bound to the membrane and band 3 clusterization (Fig. 5c, d); and (iv) increased release of erythroid microparticles (Fig. 5e). Taken together these data summarized the main findings characterizing the drug induced acute hemolysis in subjects with G6PD deficiency, supporting the role of Fyn as key regulator of G6PD activity in red cells [19,37].

**Fig. 6:**
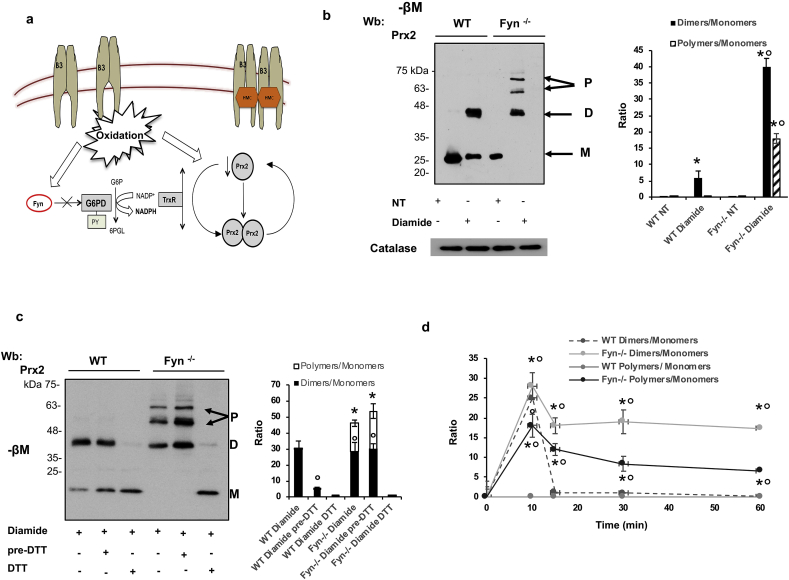
**Fyn**^**-/-**^**mouse red cells exposed to oxidation display accumulation of reduced Prx2 as dimers and polymers**. **(a)** In red cells, we propose that Fyn acts as redox sensor, modulating intracellular response to oxidation by phosphorylation of G6PD, which contributes through NADP-NADPH to thioredoxin reductase activity and Prx2 recycling. This reduces hemoglobin oxidation and the generation of reactive oxygen species (ROS), preventing the generation and translocation to the membrane of hemichromes (HMCs) that promotes band 3 (B3) clusterization and hemolysis. **(b)** Western-blot (Wb) analysis under non-reducing condition (-βM: β-mercaptoethanol) with specific antibodies against peroxiredoxin-2 (Prx2) of red cell cytosol fractions from wild-type (WT) and Fyn^-/-^ mice with or without diamide (NT: not-treated). Prx2 monomers (M), dimers (D) and polymers (P) were detected. Catalase was used as loading control. One representative gel from 4 with similar results is presented. Densitometric analysis of immunoblots is shown in bar graph on the right. Data are expressed as Dimers/monomers or Polymers/monomers ratio. Results are presented as means ± SD (*n* = 4 from each strains); *p<0.05 compared to non-treated cells; °p<0.02 compared to WT red cells. **(c)** Western-blot (Wb) analysis under non-reducing condition (-βM: β-mercaptoethanol) with specific antibody against peroxiredoxin-2 (Prx2) of wild-type (WT) and Fyn^-/−^mouse red cell cytosol fractions exposed to diamide and treated with DTT either before (Pre-DTT) or during (DTT) oxidation. Prx2 monomers (M), dimers (D) and polymers (P) were detected. Twin gels stained with colloidal Commassie were used as protein loading control One representative gel from 4 with similar results is presented (see Fig. 5Sb). Densitometric analysis of immunoblots is shown in bar graph on the right. Data are expressed as Dimers/monomers or Polymers/monomers ratio. Results are presented as means ± SD (*n* = 4 from each strains); *p<0.05 compared to WT; °p<0.02 compared to diamide treated red cells. **b-c.** *p<0.05 compared to WT; °p<0.02 compared to diamide treated red cells by two-way ANOVA with Bonferroni correction for multiple comparisons. **(d)** Recycling of Prx2 dimers and polymers in cytosol fraction from red cells of wild-type (WT) and Fyn^-/−^mice exposed to diamide and analyzed at baseline (0) and at 15, 30, 60 min after diamide incubation. Prx2 monomers (M), dimers (D) and polymers (P) were detected on immunoblots and Densitometric analysis was carried out to determine Dimers/monomers or Polymers/monomers ratio. Results are presented as means ± SD (*n* = 4 from each strains); *p<0.05 compared to WT by two-way ANOVA test with Bonferroni correction for multiple comparisons; °p<0.02 compared to baseline by one-way ANOVA with Dunnet’s test for longitudinal comparison. (For interpretation of the references to colour in this figure legend, the reader is referred to the Web version of this article.)

To further understand whether the absence of Fyn might affect a NADPH dependent anti-oxidant system such as Prx2, which is the third most abundant protein in red cells, we analyzed Prx2 monomers/dimers equilibrium in red cells from *in vivo* primaquine treated mice. As shown in Fig. 5f, the amount of dimers of Prx2 was higher in primaquine treated Fyn^-/-^ mice than in wild-type animals, indicating the accumulation of reduced Prx2 similarly to that described in patient with G6PD deficiency (Fig. 6Sa) [6].

### 3.6 Defective Prx2 recycling characterized Fyn^-/-^ mouse red cells

We thus hypothesized that in Fyn^-/-^ mouse red cells the defective G6PD activation might affect Prx2 recycling (Fig. 6a). As shown in Fig. 6b, diamide promoted dimerization of Prx2 in wild-type red cells; whereas, we observed generation of dimers/polimers with disappearance of monomers in diamide treated Fyn^-/-^ mouse red cells. The LC MS/MS analysis of the corresponding bands in the colloidal Commassie stained gels following tryptic digestion confirmed the identification of Prx2 in the selected bands (Table 3S). We then studied the effect of DTT on Prx2 dimerization in Fyn^-/-^ mouse red cells exposed to diamide. As shown in Fig. 6c, DTT prevented the diamide induced Prx2 dimerization in wild-type erythrocytes and the generation of Prx2 dimers/polimers in Fyn^-/-^ mouse red cells. It is of interest to note that the beneficial effect of DTT disappeared in red cells treated with DTT before diamide incubation (Fig. 6c: lane 2 for wild-type and lane 5 for Fyn^-/-^ mouse red cells treated with DTT before diamide incubation, Fig. 6Sb). To explore the efficiency of the recycle of reduced Prx2, we evaluated Prx2 monomers/dimers equilibrium in the same time frame used for the analysis of regeneration of GSH (see Fig. 4i). In wild-type red cells, Prx2 dimers diminished at 60 min after diamide exposition, whereas no significant change in the amounts of dimers was observed in Fyn^-/-^ mouse red cells exposed to diamide (Fig. 6d). These data indicate that the recycling of reduced Prx2 requires an efficient G6PD function to protect red cells against oxidative stress.

### 3.7 G6PD deficiency human red cells show an impairment of reduced Prx2 recycling

**Fig. 7:**
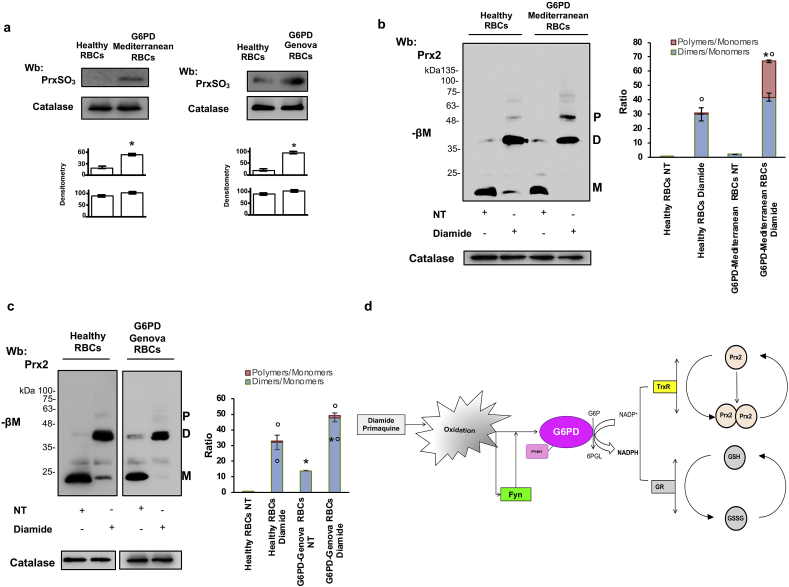
**Reduced Prx2 recycling in response to oxidation characterized red cells from G6PD Mediterranean and Genova patients**. **(a)** Western-blot (Wb) analysis with specific antibody against peroxiredoxin SO_3_ of cytosol fractions from diamide treated red cells of healthy and G6PD-Mediterranean and G6PD-Genova subjects. Catalase was used as loading control. One representative gel from 5 with similar results is presented. **Lower panel.** Densitometric analysis of immunoblots is shown in bar graph. Data are presented as means ± SD (*n* = 5 from each strains); *p<0.05 compared to healthy red blood cells by Student’s t-test. **(b)** Western-blot (Wb) analysis under non-reducing condition (-βM: β-mercaptoethanol) with specific antibody against peroxiredoxin-2 (Prx2) of red cell cytosol fractions from healthy and G6PD deficient subjects treated with or without diamide (NT: not-treated). Prx2 monomers (M), dimers (D) and polymers (P) were detected. Catalase was used as loading control. One representative gel from 6 with similar results is presented. Densitometric analysis of immunoblots is reported on the right. Dimers/monomers or Polymers/monomers ratio. Results are presented as means ± SD (*n* = 5); *p<0.05 compared to healthy red blood cells; °p<0.02 compared to NT by two-way ANOVA test with Bonferroni correction for multiple comparisons. **(c)** Western-blot (Wb) analysis under non-reducing condition (-βM: β-mercaptoethanol) with specific antibody against peroxiredoxin-2 (Prx2) of red cell cytosol fractions from healthy and G6PD deficient subjects treated with or without diamide (NT: not-treated). Prx2 monomers (M), dimers (D) and polymers (P) were detected. Catalase was used as loading control. One representative gel from 3 with similar results is presented. Densitometric analysis of immunoblots is reported on the right. Dimers/monomers or Polymers/monomers ratio. Results are presented as means ± SD (*n* = 3); *p<0.05 compared to healthy red blood cells; °p<0.02 compared to NT by two-way ANOVA test with Bonferroni correction for multiple comparisons. **(d)** Schematic diagram on the role of Fyn as redox sensor in red cells exposed to oxidative stress. The activation of Fyn phosphorylates G6PD on Tyr 401, resulting in more efficient catalytic conformation, supporting the generation of NADP-NADPH. This is required for both GSH and Prx2 recycling to scavenge peroxides, preventing red cell lysis. (For interpretation of the references to colour in this figure legend, the reader is referred to the Web version of this article.)

To explore the relevance of these findings in human red cells, we studied erythrocytes from both healthy subjects and patients with G6PD-Mediterranean and G6PD-Genova. As shown in Fig. 7a, diamide induced accumulation of PrxSO3, the irreversible oxidized form of Prx2 in G6PD-Mediterranean. This was associated with generation of Prx2 dimers/polimers when compared to healthy erythrocytes (Fig. 7b). In G6PD-Genova, we observed accumulation of Prx2 dimers in untreated cells. This was further worsened by diamide treatment, when compared to healthy controls (Fig. 7c). This was associated with a marked reduction in thioredoxin-reductase activity in both vehicle and diamide treated G6PD-deficient red cells when compared to healthy erythrocytes, which might negatively affect Prx2 recycling (Fig. 7Sa, upper panel). In agreement we found reduced G6PD activity and decreased NADPH/NADP_tot_ level in G6PD-deficient red cells when compared to healthy erythrocyte (Fig. 7Sa, lower panel, and 7Sb). These findings further support the key role of post-translation regulation of G6PD in human red cells and the involvement of the thioredoxin/Prx2 system in the increase vulnerability of G6PD deficient red cells to oxidation.

## 4 Discussion

Here, we show for the first time that Fyn kinase is a redox sensor in red cells, coordinating the activation of G6PD in response to oxidation. We link Fyn to G6PD activity starting from the observation of increase Tyr-phosphorylation of G6PD in healthy red cells, which was lacking in G6PD-Mediterranean and G6PD-Genova erythrocytes. Few previous studies in other cell models have suggested changes in protein phosphorylation state of G6PD might activate G6PD [15,38]. Our phospho-proteomic studies allowed the identification of Tyr401 as key residue in G6PD targeted by Fyn. This is extremely interesting since Tyr 401 is phylogenetically preserved from bacteria to mice and humans, further supporting the importance of Tyr401 in G6PD function. We approached the question on the role of Ty401 in G6PD activity using molecular dynamic simulation and mutagenesis experiments. Our data indicate that Tyr401 is critical for maintaining the G6PD dimer in a proper active conformation, as witnessed by the structural destabilization and the drastic reduction of the catalytic activity resulting from the mutation of this residue to Phe. Mechanistically, Tyr401 contributes to the correct placement of one molecule of NADP, which acts as a structural co-factor stabilizing G6PD, so that another molecule of NADP in the catalytic pocket can be reduced in NADP-NADPH regeneration. Significantly, this latter process is enhanced upon oxidative stress, whereby Fyn becomes overactive and phosphorylates Tyr401, an event that strongly stimulates G6PD activity by increasing the catalytic efficiency. Our data indicate that Tyr401 is important in G6PD dimer stabilization, allowing the enzyme to gain a more efficient catalytic conformation. This empowers G6PD to ensure NADP-NADPH regeneration in presence of oxidative stress. In G6PD deficient patients, mutations between 380 and 410 aa are grouped in Class I G6PD mutations, characterized by chronic non-spherocytic hemolytic anemia [[1], [2], [3],^9^]. Fyn^-/-^ mice present a moderate chronic non spherocytic hemolytic anemia, worsened by primaquine mediated oxidative stress, similarly to human subjects with Class I G6PD mutations. Taken together these data further support the importance of this protein area for G6PD function. Although the sample size of human subjects with G6PD deficiency is small and limited to one form of G6PD class I mutations, our data support the importance of Tyr-401 in modulation of G6PD activity by Fyn. Indeed, the hematologic similarities between the diamide induced misshaped Fyn^-/-^ mouse red cells associated with Hb oxidation and the blood smears from patients with G6PD deficiency during acute hemolytic crisis functionally connect Fyn and G6PD [28,37]. The specificity of G6PD as substrate of Fyn is supported by the evidence that mice genetically lacking Lyn display a preserved G6PD activation in response to oxidative stress compared to Fyn^-/-^ mouse red cells. Indeed, the delayed in GSH regeneration observed in Fyn^-/-^ mouse red cells exposed to diamide, support the role of Fyn in modulation of G6PD function, indirectly affecting NADP dependent anti-oxidant systems.

Our data also highlight the crucial functional connection between G6PD activity and the function of Prx2, the third most abundant protein and NADPH dependent anti-oxidant system. In healthy cells, Fyn acts as redox sensor, modulating intracellular response to oxidation by phosphorylation of G6PD, which contributes through NADP-NADPH to thioredoxin reductase activity and Prx2 recycling (Fig. 6a). In Fyn^-/-^ mice, the inability to support this pathway amplifies the oxidative damage, promoting the generation of hemichromes, band 3 clusterization and the release of erythroid microparticles to clear oxidized Hb and irreversible damaged proteins. Indeed, in Fyn^-/-^ mice the acute intravascular hemolytic crisis induced by primaquine associated with the accumulation of reduced Prx2 indicate the key role of Fyn in activation of G6PD. Data on red cells from G6PD patients exposed to diamide and showing higher Fyn activation than in healthy controls and accumulation of reduced and overoxidized Prx2 further supports the role of Fyn as redox sensor targeting G6PD to ensure red cell survival against oxidation.

The delayed recycling of the reduced Prx2 in oxidized red cells from both Fyn^-/-^ mice and G6PD deficient subjects indicate Prx2 as NADPH dependent anti-oxidant additional to glutathione peroxidase preventing hemolysis induced by oxidation.

In conclusion, our data contribute in the progress on the knowledge of mechanisms involved in the regulation of G6PD, a key enzyme during red cell lifespan. We show that Fyn acts as redox sensor, specifically targeting G6PD to protect GSH and Prx2, which are required to remove peroxides that contribute to hemolysis in individuals with G6PD deficiency.

## 5 Conclusions

Here, we firstly identify post-translation change in G6PD, namely Tyr-phosphorylation of residue Tyr401, which is specifically target by Fyn in both mouse and human red cells. We demonstrate that Fyn targets the phylogenic preserved Tyr 401 on C-terminus of G6PD and we confirmed by mutagenesis approach that the catalytic efficiency of G6PD towards NADP+ and G6P is strongly increased by Tyr-phosphorylation. We document that red cells from Fyn^-/−^mice are sensitive to primaquine induced intravascular hemolysis similar to that observed in G6PD deficient patients. The biologic importance of Tyr401is supported by the severity of hematologic phenotype of G6PD deficient patients, carrying mutations between 380 and 410 aa [1,3]. Although we cannot exclude Tyr-phosphorylation of other residues on G6PD, phosphorylation of Tyr-401 was the only one identified by mass spectrometric analysis ad validated by mutagenesis studies. Other Tyr residues such as Tyr503 or Tyr507 have been identified in G6PD from other cell models [15,16]. However, this protein area is not interested by mutations responsible clinically expressed G6PD deficiency [9].

Finally, our data contribute to the demonstration that the recycling of peroxiredoxin-2 (Prx2), a NADPH dependent anti-oxidant and the third most abundant protein in red cells, is decreased in Fyn^-/-^ mouse red cells with defective G6PD activation. The delayed recycling of the reduced Prx2 in oxidized red cells from both Fyn^-/-^ mice and G6PD deficient subjects indicate Prx2 as NADPH dependent anti-oxidant additional to glutathione peroxidase preventing hemolysis induced by oxidation.

## Authors contribution

MA and TE carried out the experiments, designed the study and contributed in writing the paper; FL carried out the experiments and worked on Fyn^-/-^ mice; DPML carried out the enzymatic studies; AC carried out the mass spectrometric analysis, PP analyzed proteomic data and contributed in writing the paper; CL carried out the 3D structural analysis, AMB, LDF designed the experiments, analyzed the data, wrote the manuscript; FT and AP carried out GSH studies and wrote the paper, GMS and MQJ carried out preliminary experiments on mutants for G6PD; SYC and KDW generated the G6PD WT and mutants and carried out chemic characterization; XA and MDC discussed data and contributed in writing the manuscript; EF contributed to immunoblot analysis, GLF selected the G6PD-Genova variant and contributed in data discussion.

## Authors disclosure

The Authors have nothing to disclose.

## Figures and Tables

**Fig. S1:**
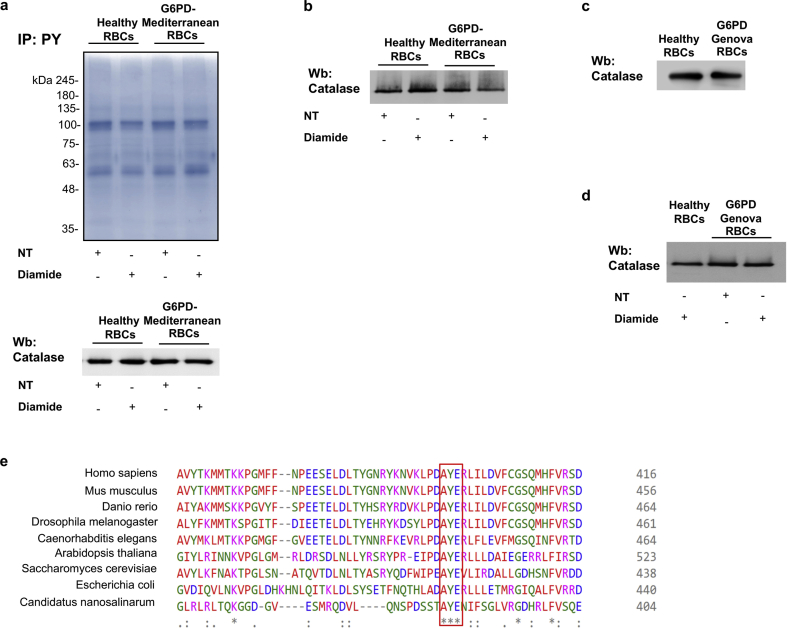


**Fig. S2:**
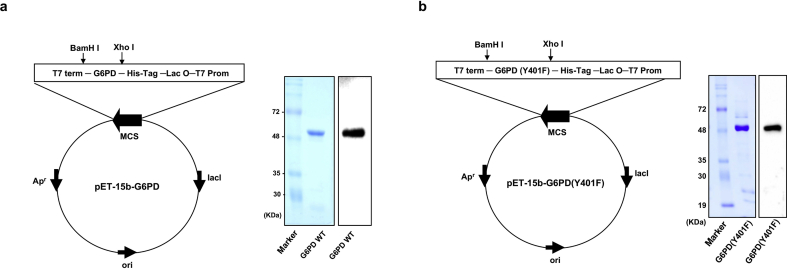


**Fig. S3:**
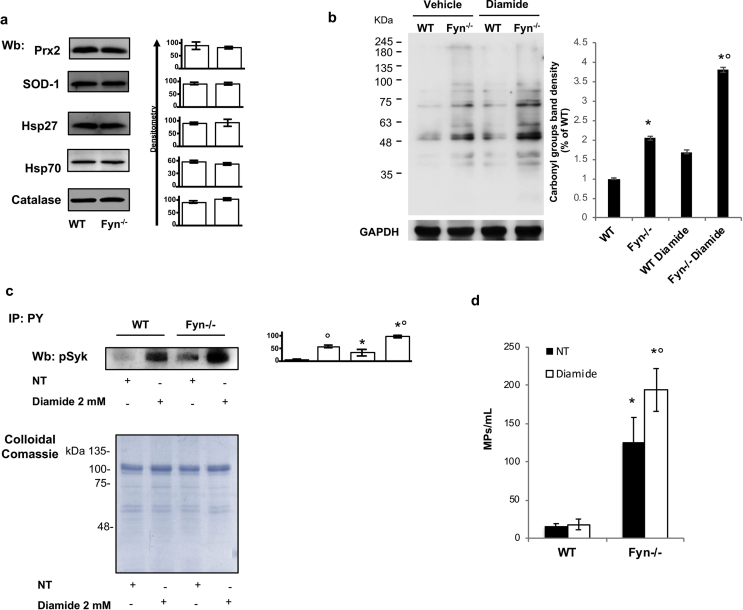


**Fig. S4:**
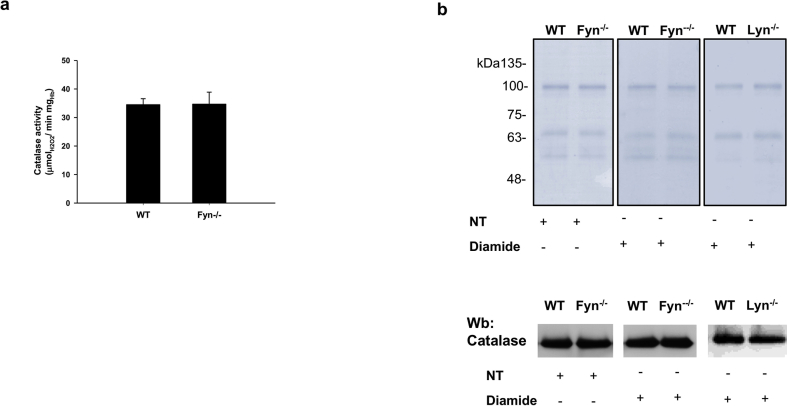


**Fig. S5:**
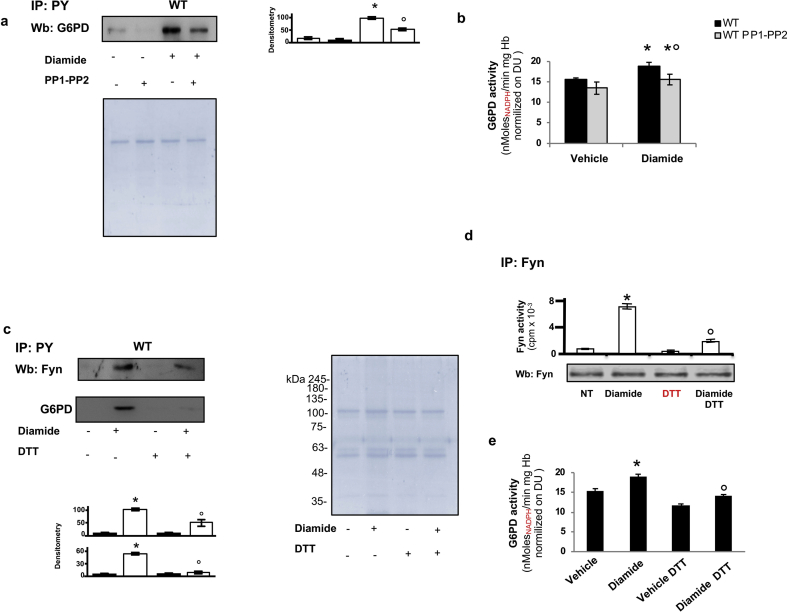


**Fig. S6:**
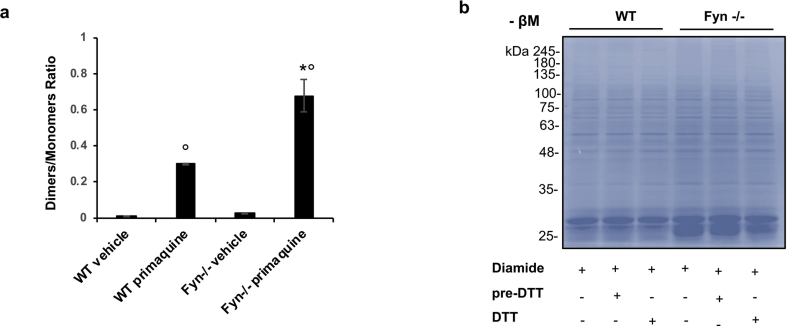


**Fig. S7:**