# Supplementary material for: Fyn specifically Regulates the activity of red cell glucose-6-phosphate-dehydrogenase
Source: Redox Biol. 2020 Jul 11;36:101639. doi: 10.1016/j.redox.2020.101639 (PMC7387845; doi:10.1016/j.redox.2020.101639)

Figure 1R (main replicates for Figure 1)

Main replicates-Figure 1a

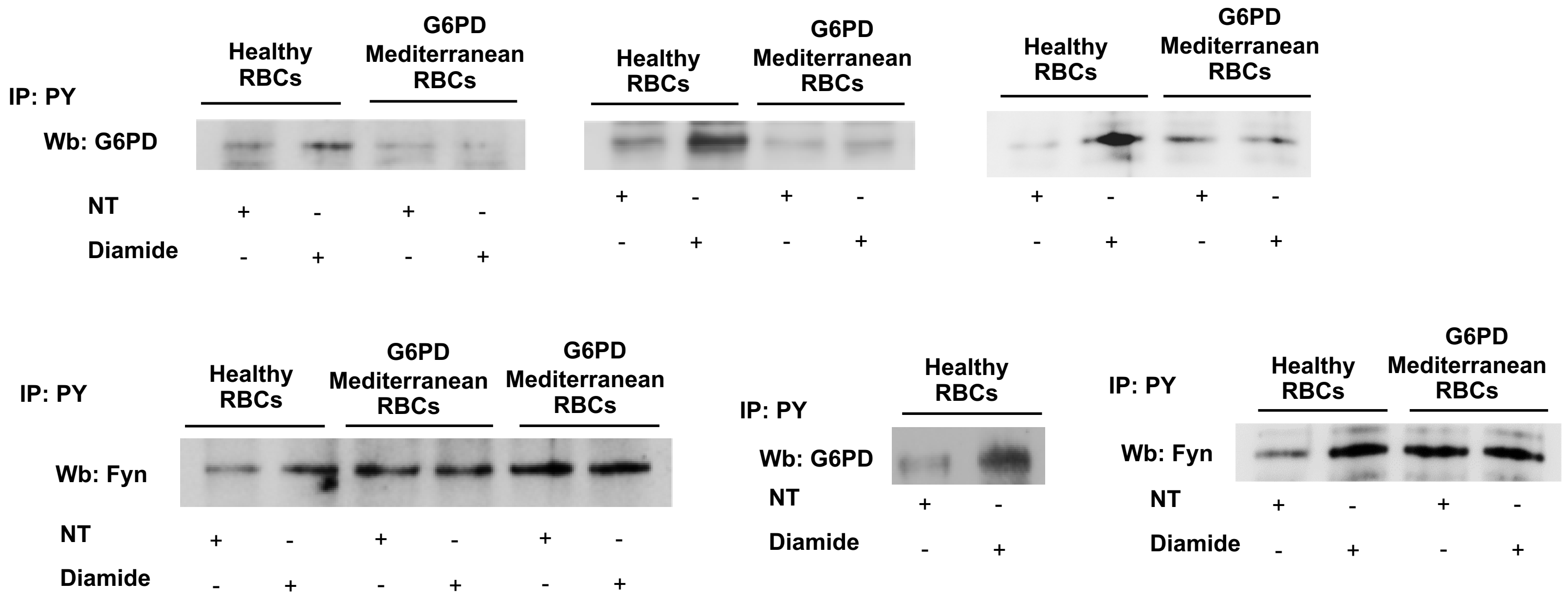

Main replicates-Figure 1b

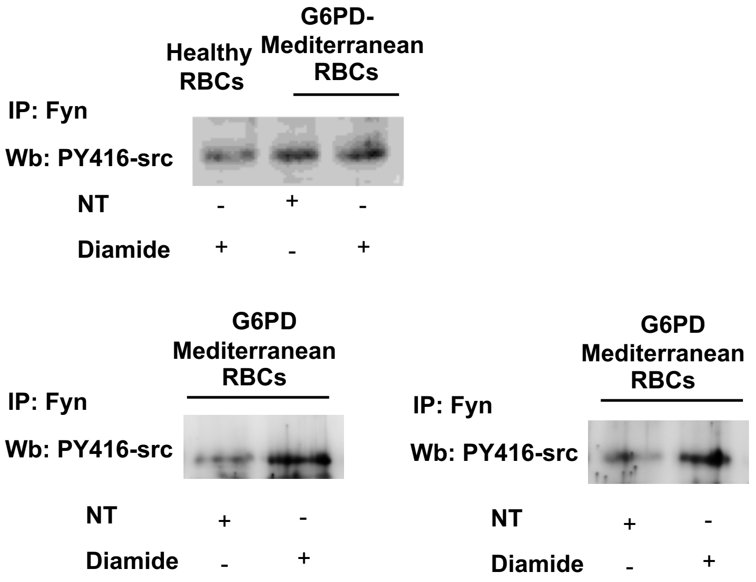

Main replicates-Figure 1c

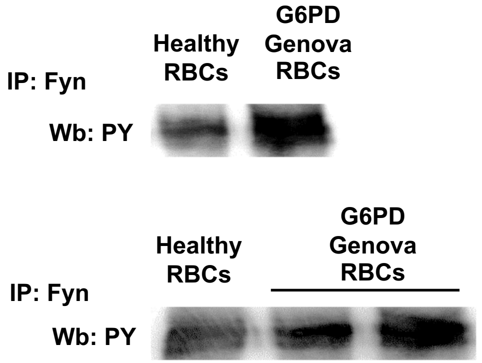

Main replicates-Figure 1d

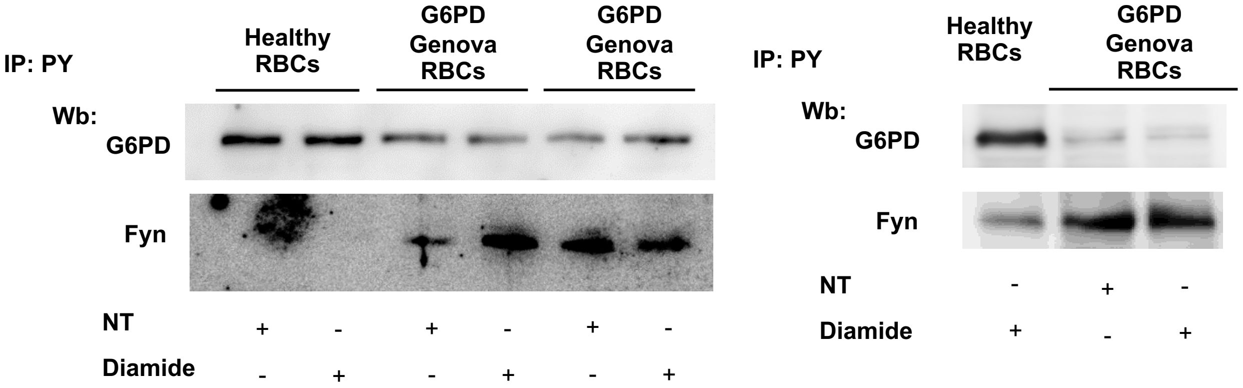

Main replicates-Figure 1f

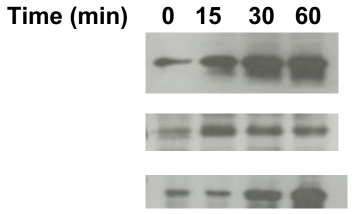

Figure 2R (main replicates for Figure 3)

Main replicates-Figure 3c

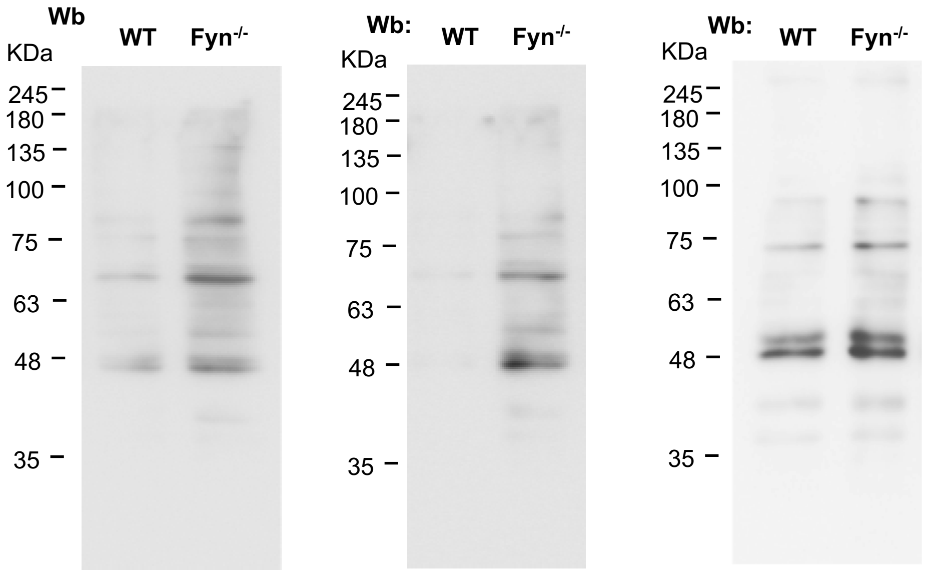

Main replicates-Figure 3e

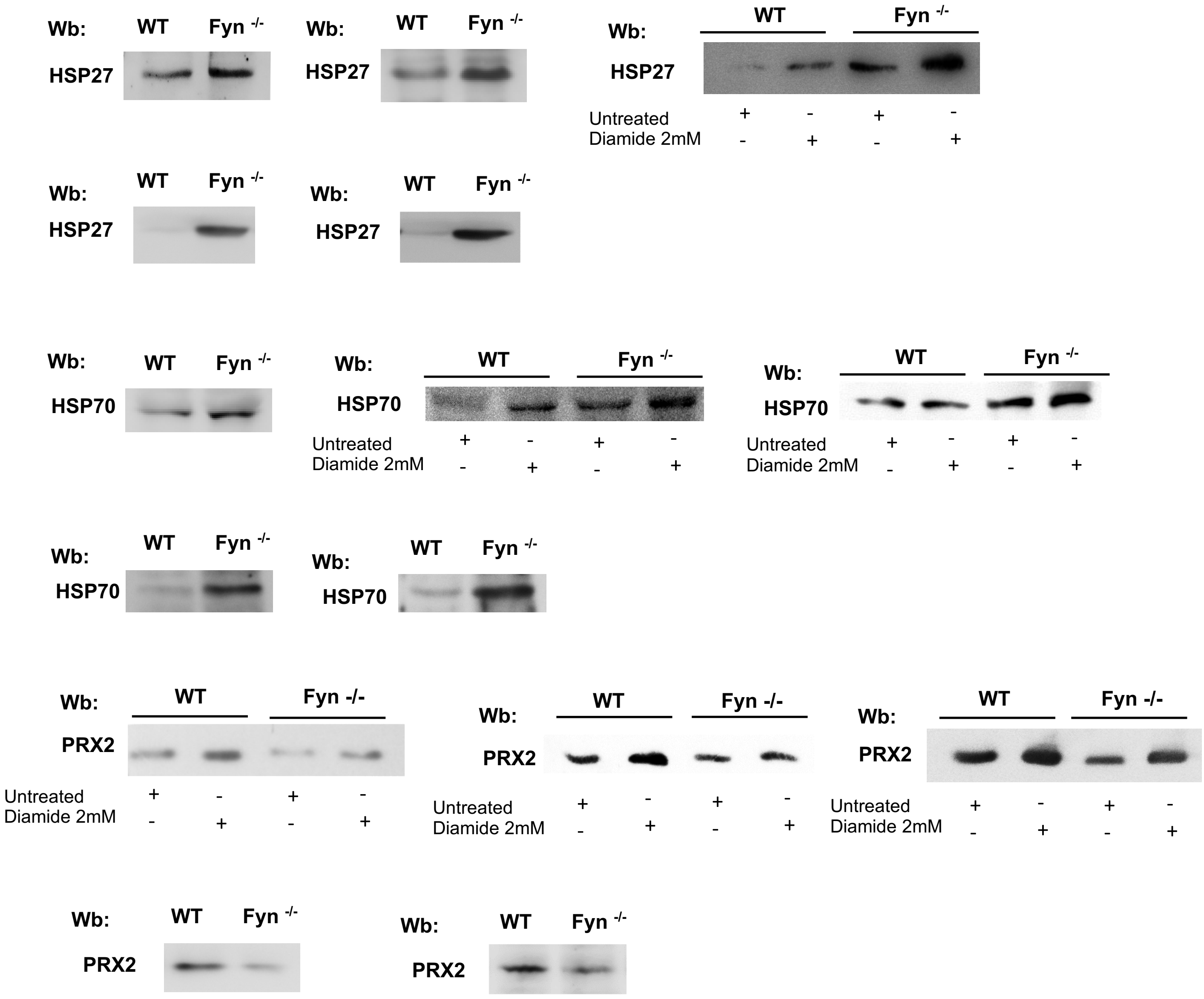

Main replicates-Figure 3f

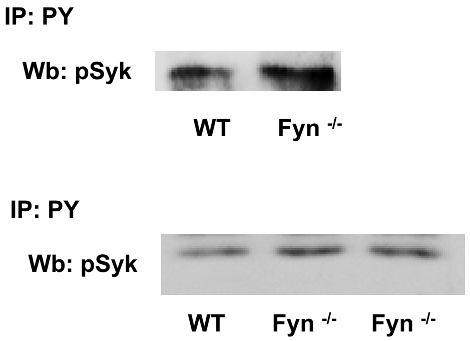

Figure 3R (main replicates for Figure 4)

Main replicates-Figure 4d

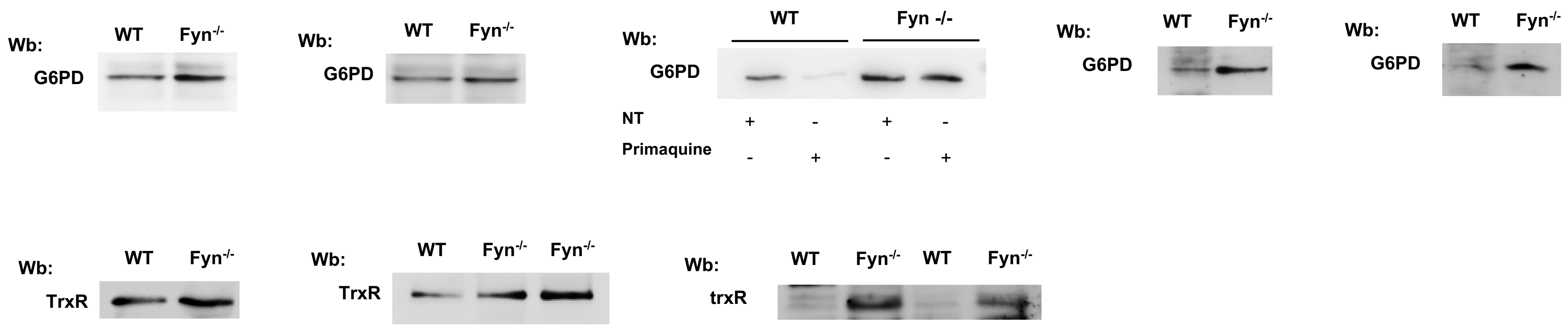

Main replicates-Figure 4g

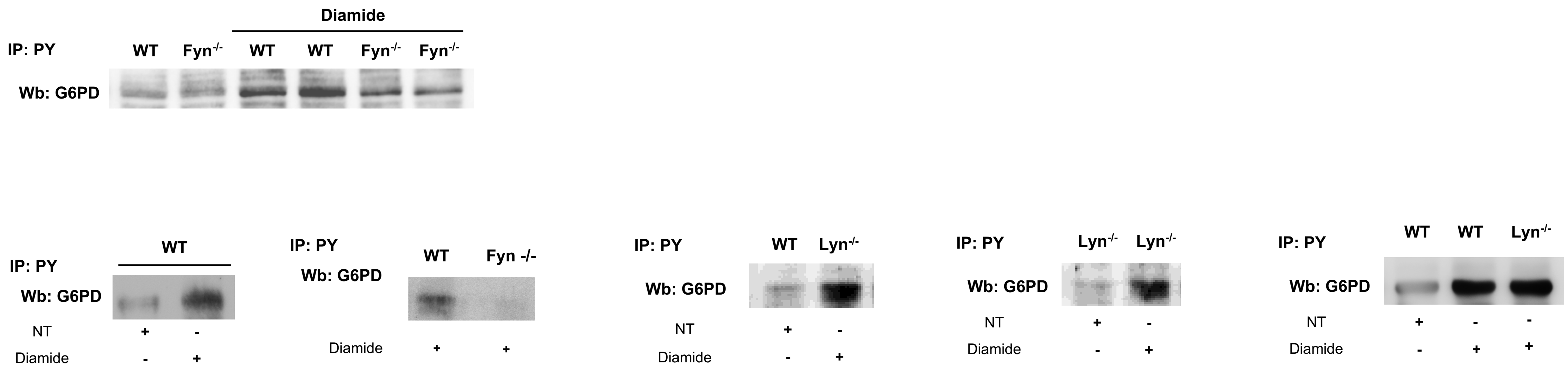

Figure 4R (main replicates for Figure 5)

Main replicates-Figure 5f

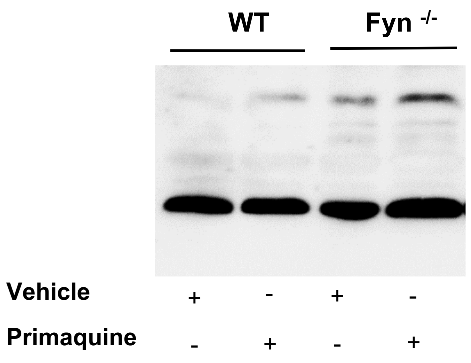

Figure 5R (main replicates for Figure 6)

Main replicates-Figure 6b

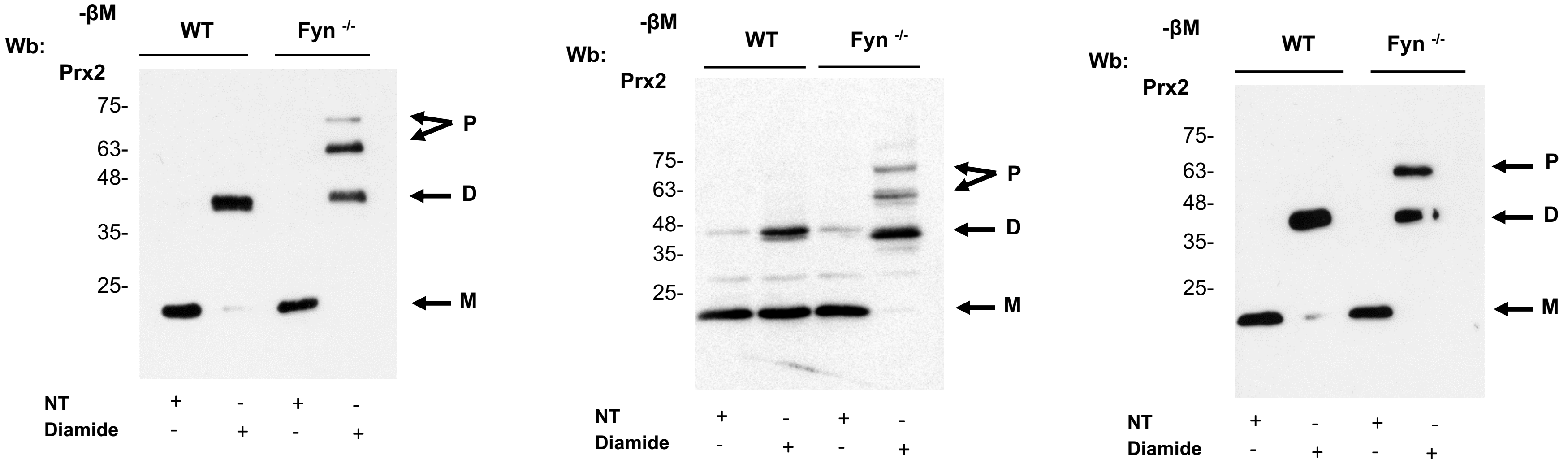

Main replicates-Figure 6c

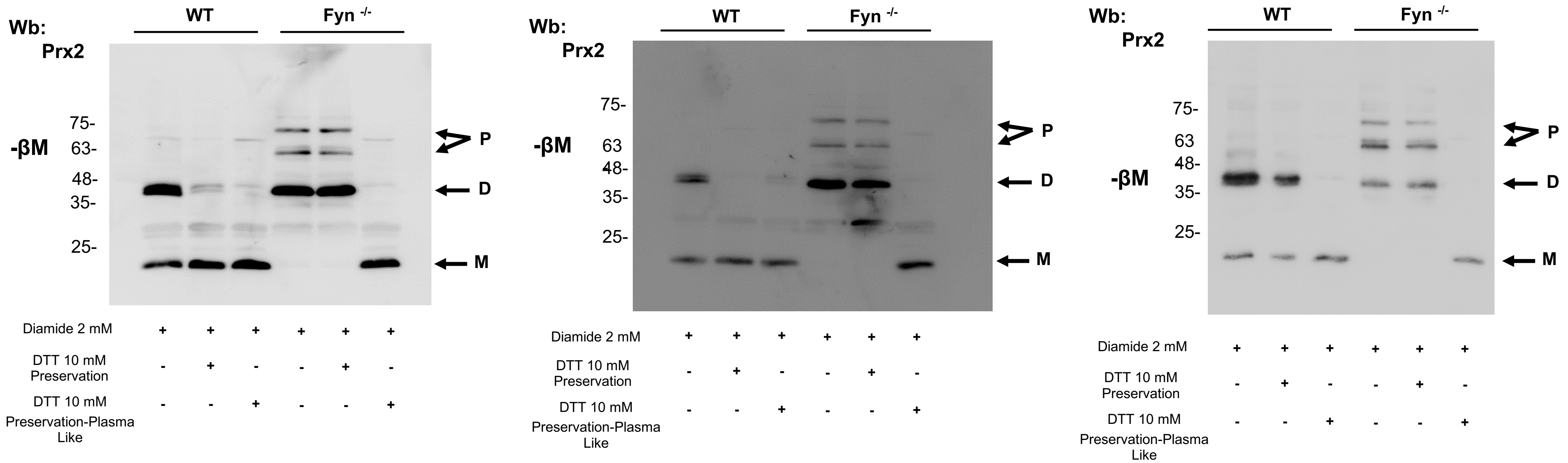

Figure 6R (main replicates for Figure 7)

Main replicates-Figure 7a

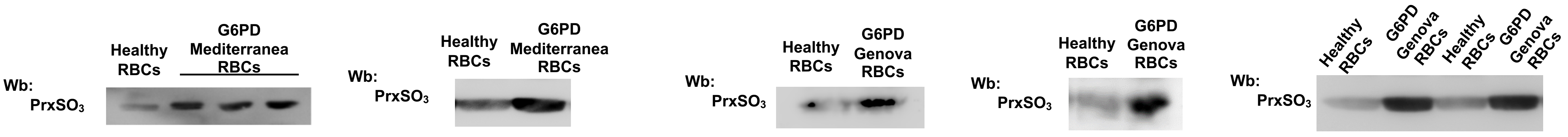

Main replicates-Figure 7b

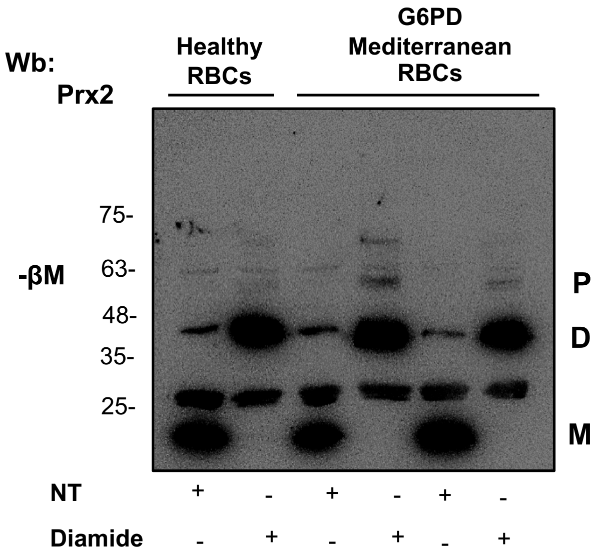

Main replicates-Figure 7c

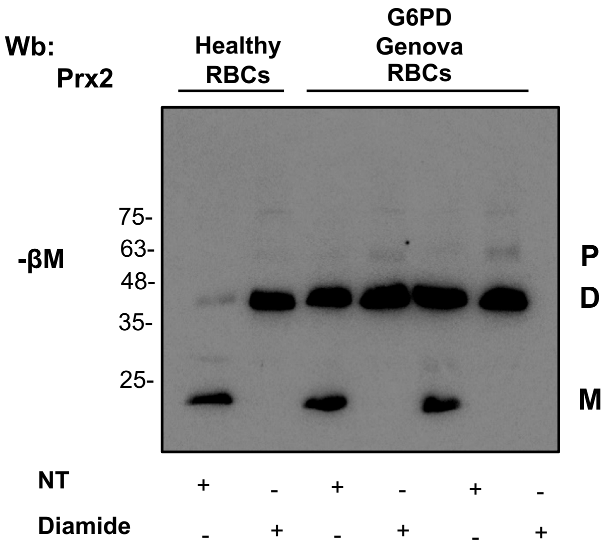

Supplement: Multimedia component 1 [file mmc1.pdf]
