# Supplementary material for: Fyn specifically Regulates the activity of red cell glucose-6-phosphate-dehydrogenase
Source: Redox Biol. 2020 Jul 11;36:101639. doi: 10.1016/j.redox.2020.101639 (PMC7387845; doi:10.1016/j.redox.2020.101639)
Supplement: Multimedia component 2 [file mmc2.docx]

| **Table 1S. Primers used in this study** | |
| --- | --- |
| **Primer Name** |  |
| Tyr401Phe fw | 5´-CAACGAGGCCGTGT**T**CACCAAGATGATGAC-3´ |
| Tyr401Phe rev | 5´-GTCATCATCTTGGTG**A**ACACGGCCTCGTTG-3´ |
| Tyr401Glu fw | 5´-CAACGAGGCCGTG**G**A**A**ACCAAGATGATGAC-3´ |
| Tyr401Glu rev | 5´-GTCATCATCTTGGT**T**T**C**CACGGCCTCGTTG-3´ |
| Flanking *NdeI* fw | 5´-CGACAGC**CATATG**GCAGAG-3 |
| Flanking *Bpu* rev | 5´-TGCGCTGA**GCTCAG**AGCTT-3 |
| The letter in bold and underline indicates the mutagenic site (see also Gomez-Manzo S et al 2014). | |

| **Table 2S. Hematological Parameters and Red Cell Indices in Wild-type and Fyn^-/-^ Mice** | | |
| --- | --- | --- |
|  | **Wildtype mice**  **(*n*=10)** | **Fyn^-/-^ mice**  **(*n*=10)** |
| Hct (%) | 48.6 ± 1.2 | 46.2 $\pm$ 0.6* |
| Hb (g/dl) | 15.8 ± 0.5 | 14.1 $\pm$ 0.8* |
| MCV (fl) | 50.0 ± 0.9 | 46.4 $\pm$ 1.1* |
| MCH (g/dl) | 16.1 ± 0.4 | 14.9 $\pm$ 0.7 |
| RDW (%) | 11.2 ± 0.2 | 13.4 $\pm$ 0.5* |
| Retics (10^3^ cells/uL) | 460± 38.6 | 560 $\pm$ 40.2* |
| Hct: hematocrit; Hb: hemoglobin; MCV: mean corpuscular volume; MCH: mean corpuscular hemoglobin; RDW: red cell distribution width; Retics: reticulocytes; *p< 0.05 compared to wild-type mice; | | |

| **Table 3S. Protein identification** | | | | |
| --- | --- | --- | --- | --- |
| **Band** | **SwissProt code** | **Protein ID** | **Mascot Score** | **Peptides** |
| **Polimers (P)** | Q61171 | Peroxiredoxin-2 | 159 | 13 |
| **Dimers (D)** | Q61171 | Peroxiredoxin-2 | 211 | 11 |
