# Supplementary material for: Fyn specifically Regulates the activity of red cell glucose-6-phosphate-dehydrogenase
Source: Redox Biol. 2020 Jul 11;36:101639. doi: 10.1016/j.redox.2020.101639 (PMC7387845; doi:10.1016/j.redox.2020.101639)
Supplement: Multimedia component 3 [file mmc3.docx]

**SUPPLEMENTAL FIGURES**

**Figure 1S. (a)** Twin colloidal Commassie stained gels for immunoprecipitated in Figure 1a. One representative gel from other 4 with similar results is presented**. (b)** Western-blot (Wb) analysis with specific antibodies against catalase on IP supernatants as in Figure 1b. One representative gel from 6 with similar results. **(c)** Western-blot (Wb) analysis with specific antibodies against catalase on IP supernatants as in Figure 1c. One representative gel from 6 with similar results. **(d)** Western-blot (Wb) analysis with specific antibodies against catalase on IP supernatants as in Figure 1d. One representative gel from 6 with similar results. **(e)** Multiple alignment of the aminoacidic sequences flanking the Tyr401 of the human G6PD in different species, showing the fully conserved residue of tyrosine 401 (highlighted in the red box) among evolution. *(asterisk): aminoacid residue fully conserved. : (colon) indicates conservation between groups of strongly similar properties, . (period) indicates conservation between groups of weakly similar properties. Sequences, from top to bottom: Homo sapiens (Q2VF42), Mus musculus (Q00612), Danio rerio (F6NHI0), Drosophila melanogaster (P12646), Caenorhabditis elegans (Q27464), Arabidopsis thaliana (Q43727), Saccharomyces cerevisiae (P11412), Escherichia coli (P0AC53), Candidatus Nanosalinarum (G0QCT5).

**Figure 2S. (a)** A pET-15b expression vector was prepared as described previously in our laboratory. Human G6PD was amplified by PCR with two primers. The sense primer 5’-CTCGAGATGGCAGAGCAGGT-3’ contained an *Xho*I restriction site. The antisense primer 5’-GGATCCTCAGAGCTTGTGGGG-3’ contained a *Bam*HI restriction site. The resulting PCR products were ligated into the TA vector and cut with *Xho*I and *Bam*HI. Fragments were then ligated into the pET-15b expression vector to generate control G6PD. Control G6PD plasmid was transformed into *Escherichia coli* BL21 (DE3) pLysS and cultured in 0.5 mM isopropyl-$\beta$-D-thio-galactoside (Duchefa, Haarlem, the Netherlands) at 30°C for over 24 hrs. Harvested cells were lysed by sonication and Control G6PD protein was purified using a Ni^b+-^ - → Ni^2+^- nitrilotriacetic acid Sepharose affinity column and PD-10 column chromatography to generate control G6PD protein. Bovine serum albumin was used as a standard and protein concentration was measured by Bradford assay. **(b)** A pET-15b expression vector was prepared as described previously in our laboratory. Human G6PD (Y401F) mutant was amplified by PCR with two primers. The sense primer 5’-CTCGAGATGGCAGAGCAGGT-3’ contained an *Xho*I restriction site. The antisense primer 5’-GGATCCTCAGAGCTTGTGGGG-3’ contained a *Bam*HI restriction site. The resulting PCR products were ligated into the TA vector and cut with *Xho*I and *Bam*HI. Fragments were then ligated into the pET-15b expression vector to generate control G6PD (Y401F). Control G6PD (Y401F) plasmid was transformed into *Escherichia coli* BL21 (DE3) pLysS and cultured in 0.5 mM isopropyl-$\beta$-D-thio-galactoside (Duchefa, Haarlem, the Netherlands) at 30°C for over 24 hrs. Harvested cells were lysed by sonication and Control G6PD (Y401F) protein was purified using a Ni^b+-^ - → Ni^2+^- nitrilotriacetic acid Sepharose affinity column and PD-10 column chromatography to generate control G6PD (Y401F) protein. Bovine serum albumin was used as a standard and protein concentration was measured by Bradford assay.

**Figure 3S**. **(a)** Western-blot (Wb) analysis with specific antibodies against heat shock protein peroxiredoxin-2 (Prx2), superoxide dismutase (SOD-1), (HSP) 27 and 70, of red cell cytosol fraction from wild-type (WT) and Fyn^-/-^mice. One representative gel from 3 with similar results is presented. catalase was used as protein loading control. Densitometric analysis of immunoblots is shown in bar graph (right panel); Data are presented as means ±SD (*n*=3 from each strains). **(b)** Red cell membrane carbonylated proteins (1ug) were detected by treating with DNPH and blotted with anti-DNP antibody. GAPDH was used as protein loading control. **Lower panel.** Quantification of band area was performed by densitometry and expressed as % of wild-type. Data are expressed as means ±SD; *p<0.05 compared to WT and °p<0.02 compared to NT cells by two-way ANOVA test with Bonferroni correction for multiple comparisons. **(c)** Ghosts from wild-type (WT) and Fyn^-/-^ mouse red cells treated with or without diamide (non-treated, NT) underwent immunoprecipitation with specific anti-phosphotyrosine antibodies (IP: PY) and then used for Western-blot (Wb) analysis with specific anti-phospho-Syk (pSyk) antibody. Twin gels stained with colloidal Commassie were used as loading control. **Right panel.** Relative quantification of immunoreactivity for pSyk is presented as mean ± SD (*n*=3); *p< 0.05 compared to WT, °p<0.02 compared to NT cells two-way ANOVA test with Bonferroni correction for multiple comparisons. **(d)** Quantification of microparticles (MPs) from red cells treated with or without diamide (non-treated, NT) from wild-type (WT) and Fyn^-/-^ mice. Data are presented as means ± SD (*n*=6); *p<0.05 compared to WT, p<0.02 compared to NT cells by two-way ANOVA test with Bonferroni correction for multiple comparisons.

**Figure 4S**. **(a)** Catalase activity in wild-type (WT) and Fyn^-/-^ mouse red cells. data are presented as means ± SD (*n*=6). **(b)** Colloidal Commassie stained twin gels of immunoprecipitates from cytosol fraction of red cells with or without diamide (non-treated: NT) of wild-type (WT), Fyn^-/-^ and Lyn^-/-^ mice (see Figure 4h).

**Figure 5S**. **(a)** **Left panel.** Cytosol fraction from wild-type (WT) red cells treated with or without diamide in the presence or absence of the Src family inhibitors PP1 (10 uM) - PP2 (10 uM) underwent immunoprecipitation with specific anti-phospho-Tyrosine antibodies (IP: PY) and then used for either Western-blot (Wb) analysis with specific glucose 6 phosphate dehydrogenase (G6PD) antibody or colloidal Commassie staining for protein loading control **Right panel.** Relative quantification of immunoreactivity for G6PD. Data are presented as mean ± SD (*n*=3); *p< 0.05 compared to vehicle treated red cells; °p<0.05 compared to diamide treated red cells by two-way ANOVA test with Bonferroni correction for multiple comparisons. **(b)** Activity of glucose 6 phosphate dehydrogenase (G6PD) in red cells from wild-type (WT) and Fyn^-/-^mice with or without diamide in presence or absence of the Src family kinases inhibitors PP1-PP2. Data are presented as means ±SD, (*n*=3); *p< 0.05 compared to vehicle treated red cells and *p<0.05 compared to diamide treated red cells by two-way ANOVA test with Bonferroni correction for multiple comparisons. **(c)** Cytosol fraction from wild-type (WT) red cells treated with or without diamide (2 mM) in presence or absence of dithiothreitol (DTT, mM) underwent immunoprecipitation with specific anti-phospho-Tyrosine antibodies (IP: PY) and then used for either Western-blot (Wb) analysis with specific glucose 6 phosphate dehydrogenase (G6PD) antibody or colloidal Commassie staining for protein loading control. **Lower panel.** Relative quantification of immunoreactivity for G6PD. Data are presented as mean ± SD (*n*=4); *p< 0.05 compared to vehicle treated red cells and °p<0.05 compared to diamide treated red cells by two-way ANOVA test with Bonferroni correction for multiple comparisons. **(d)** Wild-type (WT) red cells treated with or without diamide (2 mM) in presence or absence of dithiothreitol (DTT, mM) underwent immunoprecipitation with specific anti-Fyn antibody (IP: Fyn) and then tested for the kinase activity on a specific peptide substrate. Data are presented as means ±SD; *p< 0.05 compared to vehicle treated red cells and ° p<0.05 compared to diamide treated red cells by two-way ANOVA test with Bonferroni correction for multiple comparisons. **(e)** Activity of glucose 6 phosphate dehydrogenase (G6PD) in red cells from wild-type (WT) and Fyn^-/-^mice with or without diamide (2 mM) in presence or absence of dithiothreitol (DTT, mM) Data are presented as means ±SD; *p< 0.05 compared to vehicle treated red cells and ° p<0.05 compared to diamide treated red cells by two-way ANOVA test with Bonferroni correction for multiple comparisons.

**Figure 6S. (a)** Densitometric analysis of immunoblots is shown in bar graph on the right. Data are expressed as Dimers/monomers ratio. Results are presented as means ±SD (*n*=3); *p< 0.05 compared to vehicle treated red cells and ° p<0.05 compared to diamide treated red cells by two-way ANOVA test with Bonferroni correction for multiple comparisons. **(b)** Colloidal Commassie stained twin gels from cytosol fraction of wild-type (WT) or Fyn^-/-^ mouse red cells exposed to diamide (non-treated: NT) and either pre-treated or treated with dithiothreitol (DTT) (see also Figure 6c).

**Figure 7S**. **(a)** Activity of thioredoxin reductase (TrxR, **lower panel**) and glucose 6 phosphate dehydrogenase (G6PD, **lower panel**) in red cells from healthy, G6PD-Mediterreanean and G6PD-Genova subjects treated with or without diamide. Data are presented as means ±SD (*n*=3); *p<0.05 compared to healthy red cells and °p<0.05 compared to NT erythrocytes by two-way ANOVA test with Bonferroni correction for multiple comparisons. **(b)** NADPH/NADP_total_ ratio in wild-type (WT) and Fyn^-/-^ mice. Data are presented as means ±SD (*n*=3); *p<0.05 compared to healthy red blood cells (RBCs) by two-way ANOVA test with Bonferroni correction for multiple comparisons.
